# TG-interacting factor 1 (Tgif1)-deficiency attenuates bone remodeling and blunts the anabolic response to parathyroid hormone

**DOI:** 10.1038/s41467-019-08778-x

**Published:** 2019-03-22

**Authors:** Hiroaki Saito, Andreas Gasser, Simona Bolamperti, Miki Maeda, Levi Matthies, Katharina Jähn, Courtney L. Long, Hartmut Schlüter, Marcel Kwiatkowski, Vaibhav Saini, Paola Divieti Pajevic, Teresita Bellido, Andre J. van Wijnen, Khalid S. Mohammad, Theresa A. Guise, Hanna Taipaleenmäki, Eric Hesse

**Affiliations:** 1 Molecular Skeletal Biology Laboratory, Department of Trauma, Hand and Reconstructive Surgery, University Medical Center Hamburg-Eppendorf, Martinistr. 52, D-20246 Hamburg, Germany; 2 Mass Spectrometric Proteomics Laboratory, Institute for Clinical Chemistry and Laboratory Medicine, University Medical Center Hamburg-Eppendorf, Martinistr. 52, D-20246 Hamburg, Germany; 3 Endocrine Unit, Massachusetts General Hospital, 55 Fruit St., Boston, MA 02114 USA; 4 Department of Anatomy and Cell Biology, Indiana University School of Medicine, 635 Barnhill Dr., Indianapolis, IN 46202 USA; 5 Department of Orthopedic Surgery, Mayo Clinic, 200 1st St. SW, Rochester, MN 55905 USA; 6 Division of Endocrinology, Department of Medicine, Indiana School of Medicine, 545 Barnhill Dr., Indianapolis, IN 46202 USA; 7 Present Address: Department of Molecular and Cell Biology, Boston University, School of Dental Medicine, 72 East Concord St., Boston, MA 02118 USA

## Abstract

Osteoporosis is caused by increased bone resorption and decreased bone formation. Intermittent administration of a fragment of Parathyroid hormone (PTH) activates osteoblast-mediated bone formation and is used in patients with severe osteoporosis. However, the mechanisms by which PTH elicits its anabolic effect are not fully elucidated. Here we show that the absence of the homeodomain protein TG-interacting factor 1 (Tgif1) impairs osteoblast differentiation and activity, leading to a reduced bone formation. Deletion of Tgif1 in osteoblasts and osteocytes decreases bone resorption due to an increased secretion of Semaphorin 3E (Sema3E), an osteoclast-inhibiting factor. Tgif1 is a PTH target gene and PTH treatment failed to increase bone formation and bone mass in Tgif1-deficient mice. Thus, our study identifies Tgif1 as a novel regulator of bone remodeling and an essential component of the PTH anabolic action. These insights contribute to a better understanding of bone metabolism and the anabolic function of PTH.

## Abstract (web summary)

Parathyroid hormone (PTH) is used to treat osteoporosis, but its therapeutic mechanism remains unclear. Here, the authors show that Tgif1 is a PTH target gene, and that its deletion impairs the function of osteoblasts and PTH-induced bone formation in mice.

## Introduction

Skeletal fragility is a growing medical and socioeconomic burden for aging societies^1–3^. Bones are constantly dismantled and rebuilt by matrix-resorbing osteoclasts and bone-forming osteoblasts. This well-balanced process of bone remodeling supports the maintenance of bone quality and stability^2,4^.

Osteoblasts arise from mesenchymal precursor cells and can differentiate into matrix-entrapped osteocytes^4^. Differentiation of osteoblasts is controlled by signaling pathways and regulatory factors, including T-cell factor/lymphoid enhancing factor (Tcf/Lef), Osterix (Osx/Sp7), Runt-related transcription factor 2 (Runx2), zinc finger proteins and homeodomain proteins^5–8^. Osteoblasts and osteocytes provide activation signals to osteoclasts via receptor activator of nuclear factor-κB ligand (RANKL), which binds to the RANK receptor on pre-osteoclasts and mature osteoclasts^9,10^. Osteoblasts can also provide negative signals to osteoclasts by secreting osteoprotegerin (OPG), a soluble RANKL decoy receptor, thereby inhibiting the RANKL–RANK interaction and subsequent osteoclast activation^10,11^. In addition to the RANKL/OPG system, interleukins (ILs), insulin-like growth factors (IGFs) and Ephrin signaling also participate in the osteoblast–osteoclast crosstalk^12,13^.

During aging, bone remodeling often becomes unbalanced with bone resorption exceeding bone formation. This can cause osteoporosis characterized by a decrease in bone mass and bone mineral density (BMD), and fragility fractures^3,14^. Specific osteoporosis treatment includes the reduction of bone resorption using bisphosphonates (e.g., Alendronate, Risedronate or Zoledronate) or an antibody against RANKL (Denosumab)^14^. An alternative approach is to increase bone formation, for instance, by the intermittent administration of a recombinant fragment of human parathyroid hormone containing the first 34 amino acids (hPTH 1–34, Teriparatide, hereafter referred to PTH)^2,14,15^. If administered intermittently in a pharmacological manner, PTH stimulates bone remodeling by increasing the activity of osteoblasts and osteoclasts. This leads to a net increase in bone mass and BMD, thereby reducing the fracture risk^15–17^.

Bone formation is also strongly activated by the canonical Wnt signaling pathway, which was uncovered from rare genetic diseases^7^. For instance, a gain-of-function mutation of the Wnt co-receptor low-density lipoprotein receptor-related protein 5 (LRP5) causes a high bone mass (HBM) phenotype^18,19^. Lrp5 is important for the activation of the canonical Wnt pathway, since binding of Wnt ligands to Lrp5 or 6 and frizzled co-receptors activate the signaling cascade. Once activated, β-catenin translocates to the nucleus and induces the expression of target genes, including *Axin2* and *CyclinD1*^7^. Inhibition of Lrp5 signaling can be achieved by the binding of soluble antagonists such as Dickkopf 1 (Dkk1) and Sclerostin^20^. Sclerostin, which is encoded by the *SOST* gene, is secreted by osteocytes and binds to Lrp4–6 receptors on osteoblasts, thereby antagonizing Wnt signaling^21–23^. Similar to Lrp5, mutations reducing the expression of the *SOST* gene were identified to cause HBM in sclerosteosis and Van Buchem disease^24,25^. Supported by these findings, a monoclonal anti-sclerostin antibody (Romosozumab) has been developed as a novel bone anabolic therapy, which increases bone mass and strength and decreases the fracture risk^26–29^.

The canonical Wnt pathway and the PTH pathway are not separated and PTH signaling stimulates bone formation by cross-activating the canonical Wnt signaling using various mechanisms, including the decrease of Dkk1 and sclerostin expression^30–32^. Although great advances have been made in the development of novel anti-osteoporosis drugs, it is still very important to further decipher molecular mechanisms underlying the control of physiological and pharmacologically induced bone remodeling to reach a better understanding of these processes. Here, we identify the homeodomain protein TG-interacting factor 1 (Tgif1) as a novel Wnt and PTH target gene and a crucial regulator of osteoblast function. Absence of Tgif1 impairs osteoblast differentiation in vitro and osteoblast activity and bone formation in vivo. Deletion of *Tgif1* in osteoblasts and osteocytes also decreases bone resorption due to an increased secretion of the osteoclast-inhibiting factor Semaphorin 3E (Sema3E)^33^. Although the bone anabolic function of Wnt signaling is unaffected by the absence of Tgif1, PTH treatment fails to increase bone mass in mice lacking Tgif1 in the osteoblast lineage. This is at least in part due to an incomplete decrease in the expression of the myocyte enhancer factor 2c (Mef2c) and subsequent sclerostin expression upon PTH treatment. Thus, our study identifies Tgif1 as a novel regulator of bone remodeling and an essential component of the PTH anabolic signaling. These insights contribute to the better understanding of steady state and pharmacologically activated bone remodeling.

## Results

### Deletion of *Tgif1* impairs bone remodeling

**Fig. 1:**
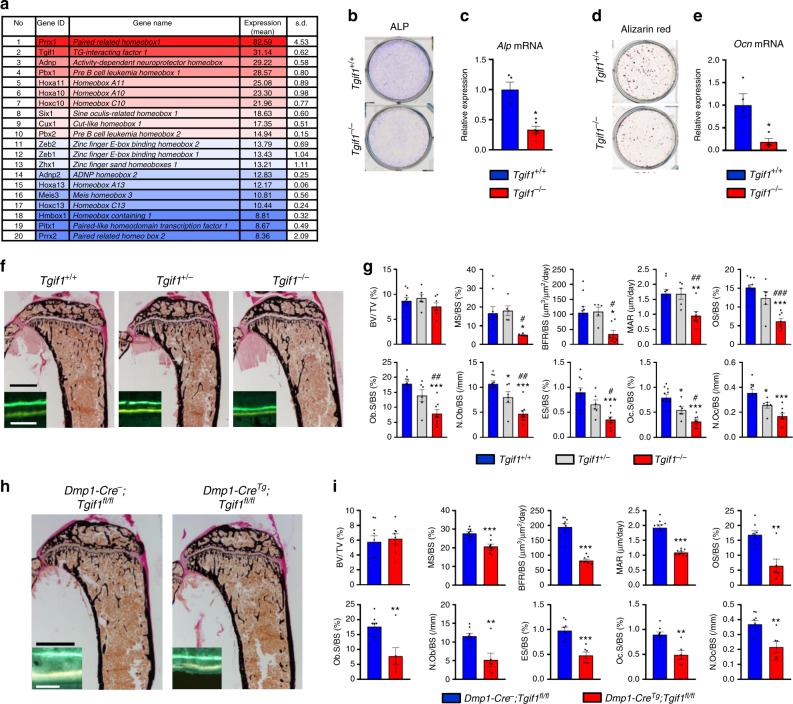
Deletion of TG-interacting factor 1 (Tgif1) in osteoblasts reduces osteoblast differentiation and bone remodeling. **a** Homeodomain proteins listed according to their expression in mouse bone marrow stromal cells (BMSCs) during osteoblast differentiation. Expression is indicated as mean values (arbitrary units) with the respective standard deviation (s.d.) (*N* = 3). The first 10 genes are marked in shades of red and the remaining 10 genes are presented in shades of blue. **b** Staining of alkaline phosphatase (ALP) activity after in vitro differentiation of *Tgif1*^*+/+*^ and *Tgif1*^*−/−*^ calvarial osteoblasts for 14 days (representative image of 3 experiments) and **c** relative *Alp* mRNA expression (*N* = 4). **d** Determination of matrix mineralization by Alizarin Red staining after 21 days of differentiation (representative image of 3 experiments) and **e** relative *osteocalcin* (*Ocn*) mRNA expression (*N* = 4). **f** Representative images of proximal tibiae of 8-week-old male mice with the genotypes *Tgif1*^*+/+*^, *Tgif1*^*+/*^^−^ and *Tgif1*^*−/−*^ after von Kossa staining and fluorescence double labeling to visualize bone formation (insets). **g** Histomorphometric analysis of the proximal tibiae of 8-week-old male *Tgif1*^*+/+*^, *Tgif1*^*+/−*^ and *Tgif1*^*−/−*^ mice (*N* = 10, 6 and 8). BV/TV bone volume/tissue volume, MS/BS mineralizing surface/bone surface, BFR/BS bone formation rate/bone surface, MAR mineral apposition rate, OS/BS osteoid surface/bone surface, Ob.S/BS osteoblast surface/bone surface, N.Ob/BS number of osteoblasts/bone surface, ES/BS eroded surface/bone surface, Oc.S/BS osteoclast surface/bone surface, N.Oc/BS number of osteoclasts/bone surface. *p<0.05, **p<0.01, ***p<0.001 vs. *Tgif1*^*+/+*^, ^#^*p*<0.05, ^##^*p*<0.01, ^###^*p*<0.001 vs. *Tgif1*^*+/−*^. **h** Representative images of proximal tibiae of 8-week-old male mice with the genotypes *Dmp1- Cre*^*−*^*;Tgif1*^*fl/fl*^ and *Dmp1-Cre*^*Tg*^*;Tgif1*^*fl/fl*^ after von Kossa staining and fluorescence double labeling to visualize bone formation (insets). **i** Histomorphometric analysis of the proximal tibiae of 8-week-old male *Dmp1-Cre*^*−*^*;Tgif1*^*fl/fl*^ and *Dmp1-Cre*^*Tg*^*;Tgif1*^*fl/fl*^ mice (*N* = 9, 8). For abbreviations see **g**. **p<0.01, ***p<0.001 vs. *Dmp1-Cre-;Tgif1*^*fl/fl*^. Scale bars indicate 1  mm (black) and 50 μm (white) **f**, **h**. Error bars represent the s.e.m. Two-tailed Student's *t*-test was used to compare two groups **c**, **e**, **i**, and analysis of variance (ANOVA) followed by Newman–Keuls post-hoc analysis was used to compare three groups **g**

Homeodomain proteins are transcriptional regulators that play important roles in osteoblast differentiation and bone formation^5,6^. To uncover homeodomain proteins with a yet unknown function in the osteoblast lineage, we performed RNA-sequencing in mouse bone marrow stromal cells (BMSCs) and identified Tgif1 as the most abundantly expressed homeodomain protein of unknown function in bone (Fig. 1a). To elucidate the role of Tgif1 in bone, we obtained osteoblasts from mice with a germline deletion of *Tgif1* (*Tgif1*^*−/−*^)^34^. Using in vitro differentiation assays, we determined that loss of Tgif1 in osteoblasts decreased the alkaline phosphatase (ALP) activity (Fig. 1b) and the *Alp* expression (Fig. 1c). Furthermore, Tgif1 deficiency impaired the matrix mineralization (Fig. 1d) and reduced the *osteocalcin* expression (Fig. 1e), demonstrating that Tgif1 promotes osteoblast differentiation.

To rule out the possibility that Tgif2, a paralog of Tgif1^35^, is increased in the absence of Tgif1 in a compensatory manner, we quantified the expression of Tgif2 in *Tgif1*^*−/−*^ osteoblasts. *Tgif2* expression was lower than the expression of *Tgif1* (Supplementary Fig. 1a) and unchanged in the absence of Tgif1 (Supplementary Fig. 1a, b), suggesting that the lack of Tgif1 is not compensated by an increased expression of Tgif2.

In order to elucidate the role of Tgif1 in bone remodeling, we investigated the bone phenotype of Tgif1-deficient mice. Histomorphometric analysis revealed that the absence of one or both alleles of *Tgif1* did not change bone mass (bone volume per tissue volume (BV/TV)) in the proximal tibiae or the lumbar spine (Fig. 1f, g and Supplementary Fig. 2a, b and Supplementary Tables 1 and 2). However, osteoblast parameters including the number of osteoblasts per bone surface (N.Ob/BS), osteoblast surface per bone surface (Ob.S/BS) and functional indices like the mineral appositions rate (MAR) and the bone formation rate per bone surface (BFR/BS) were significantly decreased in *Tgif1*^*−/−*^ mice compared to control littermates (Fig. 1f insets, 1g and Supplementary Tables 1 and 2). The greatly impaired osteoblast-mediated bone formation was accompanied by a significant reduction of osteoclast parameters, including the number of osteoclasts per bone surface (N.Oc/BS) and the osteoclast surface per bone surface (Oc.S/BS) (Fig. 1g and Supplementary Tables 1 and 2). This led to a reduced eroded surface per bone surface (ES/BS) and therefore to a diminished bone resorption (Fig. 1g and Supplementary Tables 1 and 2). The combined reduction of bone formation and bone resorption caused a gender-independent low-turnover bone phenotype. Thus, these findings confirm the positive role of Tgif1 for osteoblast function and bone formation and demonstrate that Tgif1 is a novel regulator of bone remodeling.

To further investigate these findings, we targeted the deletion of *Tgif1* to the osteoblast lineage by crossing mice carrying the *Tgif1* gene flanked by loxP sites (*Tgif1*^*fl/fl*^)^34^ with mice expressing the Cre recombinase downstream of the *Osterix* (*Osx/Sp7*) promoter (*Osx-Cre*^*Tg*^)^36^ or the *Dentin matrix protein 1* (*Dmp1*) regulatory element (*Dmp1-Cre*^*Tg*^)^37^. Lack of Tgif1 in osteoblasts in *Osx-Cre*^*Tg*^*;Tgif1*^*fl/fl*^ or in *Dmp1-Cre*^*Tg*^*;Tgif1*^*fl/fl*^ mice recapitulated the bone phenotype of *Tgif1*^*−/−*^ mice (Fig. 1h, i and Supplementary Fig. 2c–h and Supplementary Tables 3 and 4), confirming that Tgif1 in mature osteoblasts and osteocytes is required for the full activation of bone remodeling.

### Semaphorin 3E impairs osteoclast differentiation

**Fig. 2:**
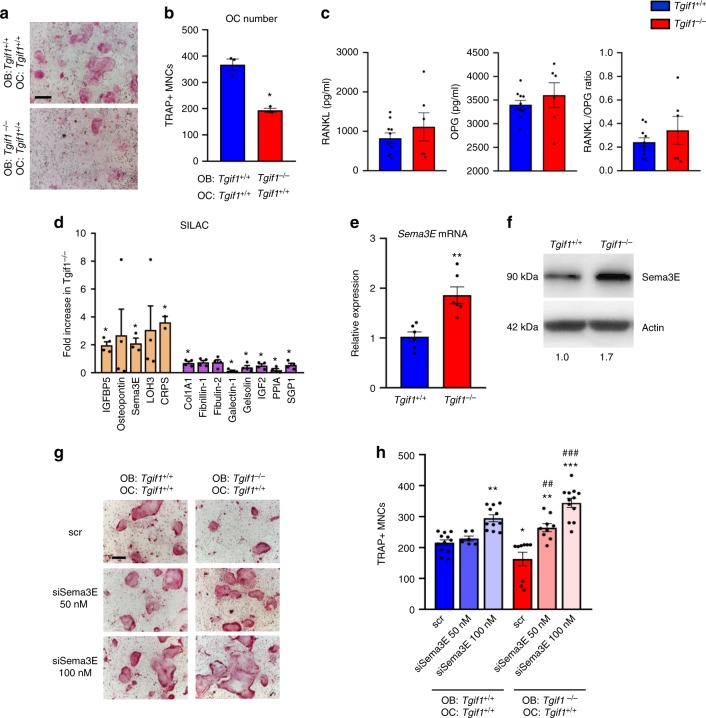
TG-interacting factor 1 (Tgif1)-deficient osteoblasts inhibit osteoclast differentiation in a Semaphorin 3E (Sema3E)-dependent manner. **a** Tartrate-resistant acid phosphatase (TRAP) staining of osteoclasts (OC) in an osteoblast–osteoclast co-culture with *Tgif1*^*+/+*^ osteoclasts and *Tgif1*^*+/+*^ or *Tgif1*^*−/−*^ osteoblasts (OB) (representative image of 3 experiments). **b** Quantification of TRAP-positive multinucleated cells (MNCs) with a minimum of three nuclei (*N* = 3). **c** Serum concentration of RANKL (receptor activator of nuclear factor NF-κB ligand) and OPG (osteoprotegerin) in *Tgif1*^*+/+*^ and *Tgif1*^*−/−*^ mice measured by enzyme-linked immunosorbent assay (ELISA) and calculation of the RANKL/OPG ratio (*N* = 10, 6). **d** Fold increase in proteins secreted by *Tgif1*^*−/−*^ calvarial osteoblasts relative to *Tgif1*^*+/+*^ calvarial osteoblasts as determined by stable isotope labeling by amino acids in cell culture (SILAC; *N* = 4). **e** Relative *Sema3E* mRNA expression in calvarial osteoblasts harvested from *Tgif1*^*+/+*^ and *Tgif1*^*−/−*^ mice (*N* =6, 6). **b**, **d**, **e** *p<0.05 **p<0.01 vs. *Tgif1*^*+/+*^ osteoblasts. **f** Immunoblot of Sema3E protein expression in calvarial osteoblasts obtained from *Tgif1*^*+/+*^ and *Tgif1*^*−/−*^ mice. Immunoblot for Actin was used as a loading control. Normalized fold expression and molecular weight in kilo Dalton (kDa) are indicated (representative image of 4 experiments). **g** Osteoclast differentiation in a co-culture system with *Tgif1*^*+/+*^ OC and *Tgif1*^*+/+*^ and *Tgif1*^*−/−*^ OB transfected with scrambled (scr) control siRNA or Sema3E siRNA (siSema3E) at indicated concentrations (representative image of 4 experiments) and **h** quantification of TRAP-positive MNCs (*N* = 12). *p<0.05, **p<0.01, ***p<0.001 vs. *Tgif1*^*+/+*^ scr., ^##^*p*<0.01, ^###^*p*<0.001 vs. *Tgif1*^*−/−*^ scr. Scale bars indicate 100 μm **a**, **g**. Error bars represent the s.e.m. Two-tailed Student's *t*-test was used to compare two groups **b**–**e**, and analysis of variance (ANOVA) followed by Newman–Keuls post-hoc analysis was used to compare more than two groups **h**

Interestingly, histomorphometric analysis of bones from mice bearing an osteoblast-targeted deletion of *Tgif1* revealed a suppression of osteoclast differentiation and bone resorption (Fig. 1i and Supplementary Fig. 2d and Supplementary Table 3 and 4), suggesting a Tgif1-dependent signaling between osteoblasts and osteoclasts. Indeed, in an in vitro co-culture system, *Tgif1*^*−/−*^ osteoblasts were impaired to support osteoclast differentiation demonstrated by a reduced number of tartrate-resistant acid phosphatase (TRAP)-positive multinucleated cells (Fig. 2a, b).

Osteoblast-derived factors are well established to contribute to the differentiation and function of osteoclasts^12,38,39^. The most prominent system includes RANKL, which emerges from osteoblasts and binds to its receptor RANK on osteoclasts and can be antagonized by OPG^9,10^. We therefore quantified the serum concentration of RANKL and OPG in *Tgif1*^*−/−*^ and *Tgif1*^*+/+*^ mice, and calculated the resulting RANKL/OPG ratio but did not detect any differences (Fig. 2c). In addition, the expression of *Rankl* and *Opg* was identical in tibiae, calvarial osteoblasts and in long bone osteoblasts from *Tgif1*^*−/−*^ mice and control littermates with no change in the *Rankl*/*Opg* ratio (Supplementary Fig. 3a–c). Next, we investigated the expression of genes encoding additional factors known to regulate the osteoblast–osteoclast interaction, including *Ephrin type-B receptor 2* and *4* (*Eph2B*, *Eph4B*), *Ephrin 2B*^40^, *ILs* (*IL-1*, *IL-6*, *IL-11*, *IL-18*)^41^, *IGFs* (*IGF-1*, *IGF-2*) and of their respective binding proteins (*Igfbp-2*, *Igfbp-3*, *Igfbp-5*)^13^ but did not determine an altered expression in *Tgif1*^*−/−*^ mice (Supplementary Fig. 3d). In order to identify the secreted factor by which *Tgif1*^*−/−*^ osteoblasts suppress osteoclast differentiation, we performed an unbiased secretome analysis using stable isotope labeling by amino acids in cell culture (SILAC). Among the factors that were significantly increased in *Tgif1*^*−/−*^ osteoblasts (Fig. 2d), we focused on Sema3E, a class III semaphorin previously shown to inhibit osteoclast formation^33^. Gene expression and immunoblot analysis confirmed a higher expression of Sema3E in *Tgif1*^*−/−*^ osteoblasts (Fig. 2e, f), suggesting that Tgif1 inhibits Sema3E expression to allow normal osteoclast differentiation. Indeed, small interfering RNA (siRNA)-mediated inhibition of Sema3E expression in *Tgif1*^*−/−*^ osteoblasts (Supplementary Fig. 3e) restored the differentiation of co-cultured osteoclasts to the level of control (Fig. 2g, h), demonstrating that Tgif1 deficiency increases the expression of the osteoblast–osteoclast signaling molecule Sema3E, leading to an inhibition of osteoclast differentiation.

### Tgif1 deficiency in bone does not alter Wnt signaling

**Fig. 3:**
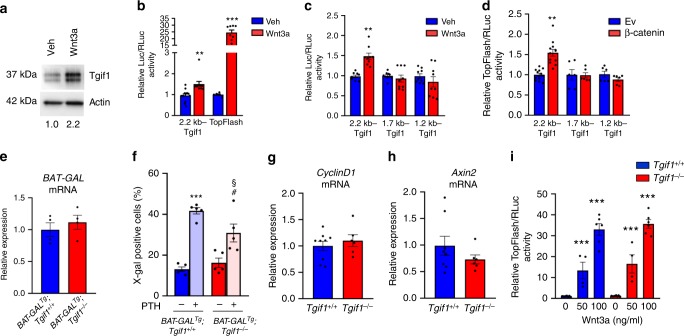
Deletion of TG-interacting factor 1 (Tgif1) does not alter canonical Wnt signaling. **a** Immunoblot of Tgif1 protein expression in wild-type calvarial osteoblasts after stimulation with recombinant Wnt3a or vehicle (Veh) for 4 h. Immunoblot for Actin was used as a loading control. Normalized fold expression and molecular weight in kilo Dalton (kDa) are indicated (representative image of 4 experiments). **b** Luciferase assays in wild-type calvarial osteoblasts transfected with a 2.2 kb Tgif1 reporter construct or a Wnt-responsive TopFlash reporter plasmid and stimulated with Veh or Wnt3a for 24 h. **c** ST2 cells were transfected with a 2.2 kb Tgif1 reporter construct and different truncations thereof, and stimulated for 4 h with Veh or Wnt3a. **d** ST2 cells were co-transfected with empty vector (Ev) or β-catenin and the 2.2 kb Tgif1 reporter construct or truncated forms thereof. **b**–**d** **p<0.01, ***p< 0.001 vs. Veh or Ev control. **e** Relative *β-galactosidase* (*BAT-GAL*) mRNA expression in tibiae of *BAT-GAL*^*Tg*^*;Tgif1*^*+/+*^ and *BAT-GAL*^*Tg*^*;Tgif1*^*−/−*^ mice (*N* = 4, 4). **f** Quantification of the fraction of β-galactosidase-positive bone marrow stromal cells (BMSCs) obtained from *BAT-GAL*^*Tg*^*;Tgif1*^*+/+*^ and *BAT-GAL*^*Tg*^*;Tgif1*^*−/−*^ mice upon stimulation with parathyroid hormone (PTH) for 4 h (*N* = 5, 5). ***p< 0.001 vs. *BAT-GAL*^*Tg*^*;Tgif1*^*+/+*^;Veh, ^#^*p*< 0.05 vs. *BAT-GAL*^*Tg*^*;Tgif1*^*−/−*^; Veh, ^§^*p*<0.05 vs. *BAT-GAL*^*Tg*^*;Tgif1*^*+/+*^;PTH. **g** Relative mRNA expression of the Wnt pathway target genes *CyclinD1* and **h** *Axin2* in tibiae of 12-week-old male mice of the genotype *Tgif1*^*+/+*^ or *Tgif1*^*−/−*^ (*N* = 8, 6). **i** Relative activation of the canonical Wnt pathway upon stimulation with increasing concentrations of recombinant Wnt3a in calvarial osteoblasts of the genotypes *Tgif1*^*+/+*^ and *Tgif1*^*−/−*^ quantified by a TopFlash reporter gene assay (*N* = 6). ***p< 0.001 vs. cells of the same genotype without Wnt3a stimulation. Error bars represent the s.e.m. Two-tailed Student's *t*-test was used to compare two groups **b**–**e**, **g**, **h**, and analysis of variance (ANOVA) followed by Newman–Keuls post-hoc analysis was used to compare more than two groups **f**, **i**

Since canonical Wnt signaling is a strong stimulator of bone formation^7^ and because Tgif1 has been implicated in oncogenic Wnt signaling, we hypothesized that Tgif1 could be an important regulator of this pathway in bone^42,43^. To test this hypothesis, we stimulated osteoblasts with Wnt3a and determined an increase in Tgif1 protein expression (Fig. 3a), thereby establishing *Tgif1* as a canonical Wnt target gene. Analysis of the 2.2 kb upstream region of the *Tgif1* promoter revealed the presence of putative Tcf/Lef binding sites, suggesting a transcriptional mechanism underlying the increase in Tgif1 expression upon Wnt stimulation. Indeed, a reporter gene assay revealed an activation of the *Tgif1* promoter in osteoblasts upon Wnt3a stimulation (Fig. 3b). Furthermore, reporter gene activation in osteoblasts by Wnt3a (Fig. 3c) or co-transfection with β-catenin (Fig. 3d) was abolished using truncated promoter fragments lacking Tcf/Lef binding sites. These findings demonstrate that *Tgif1* is a Wnt target gene.

Next, we determined if Tgif1 affects the Wnt pathway activity. To address this question, we crossed reporter mice expressing the β-galactosidase in the presence of activated β-catenin (*BAT-GAL*^*Tg*^) with *Tgif1*^*−/−*^ mice (*BAT-GAL*^*Tg*^*;Tgif1*^*−/−*^). Expression of the β-galactosidase in *BAT-GAL*^*Tg*^*;Tgif1*^*−/−*^ reporter mice as a surrogate for in vivo Wnt activity was unchanged compared to control mice, suggesting that Tgif1 might not play a functional role in Wnt signaling in bone (Fig. 3e). To ensure the functionality of the system, we harvested BMSCs from long bones of *BAT-GAL*^*Tg*^*;Tgif1*^*+/+*^ and *BAT-GAL*^*Tg*^*;Tgif1*^*−/−*^ mice and treated the cells with vehicle or PTH, which is known to cross-activate Wnt signaling^2^. Quantification of the β-galactosidase-positive cells revealed a significant induction of Wnt signaling in BMSCs of both genotypes (Fig. 3f), confirming that the pathway can be activated in this system. The lack of a regulatory role of Tgif1 in Wnt signaling in bone was further confirmed by an unchanged expression of the Wnt target genes *CyclinD1* and *Axin2* in bones of *Tgif1*^*−/−*^ mice compared to control littermates (Fig. 3g, h). Furthermore, Wnt3a activated the TopFlash reporter assay equally in *Tgif1*^*−/−*^ and control osteoblasts (Fig. 3i), indicating that in bone the canonical Wnt pathway activity is independent of Tgif1 under steady-state conditions.

### Tgif1 is dispensable for the Wnt-mediated gain in bone mass

**Fig. 4:**
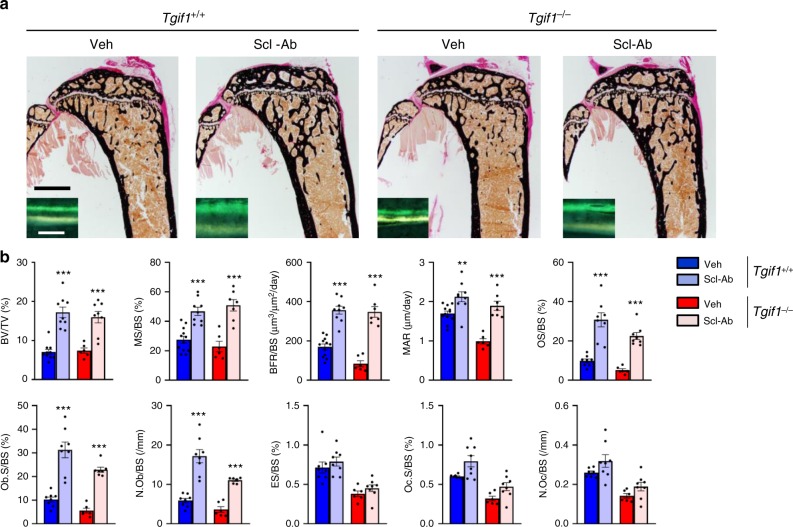
TG-interacting factor 1 (Tgif1) is dispensable for the bone anabolic function of canonical Wnt signaling. **a** Representative images of von Kossa-stained histological sections and fluorescence double labeling to visualize bone formation (insets) of the proximal tibiae of 12-week-old *Tgif1*^*+/+*^ and *Tgif1*^*−/−*^ male mice after treatment with anti-sclerostin antibody (Scl-Ab) or vehicle (Veh). Scale bars indicate 1 mm (black) and 50 μm (white). **b** Histomorphometric analysis of the proximal tibiae of 12-week-old male *Tgif1*^*+/+*^ and *Tgif1*^*−/−*^ mice after treatment with Scl-Ab or Veh (*Tgif1*^+/+^+Veh: *N* = 12, *Tgif1*^+/+^+Scl-Ab: *N* = 9, *Tgif1*^−/−^+Veh: *N* = 6, *Tgif1*^−/−^+Scl-Ab: *N* = 8). For abbreviations see the legend to Fig. 1. **p< 0.01, ***p< 0.001 vs. Veh-treated group of the same genotype. Error bars represent the s.e.m. Statistical analysis was performed using analysis of variance (ANOVA) followed by Newman–Keuls post-hoc test

To test the possibility that the absence of Tgif1 affects bone remodeling in response to Wnt activation, we injected *Tgif1*^*−/−*^ mice and control animals with an antibody against the Wnt pathway inhibitor sclerostin (Scl-Ab). Quantification of the bone mass revealed that the Scl-Ab-mediated increase in BV/TV was comparable between genotypes (Fig. 4a, b and Supplementary Table 5). Next, we crossed two knock-in mouse lines each bearing a HBM mutation of *Lrp5* that are equivalent to mutations found in humans (*G170V* and *A213V*)^44^ with *Tgif1*^*−/−*^ animals to constitutively activate Wnt signaling. Consistently, the gain in bone mass in *Lrp5*^*G170V/+*^ and *Lrp5*^*A213V/+*^ mice was independent of Tgif1 (Supplementary Tables 6 and 7). However, a detailed histomorphometric analysis uncovered that activation of the Wnt pathway in response to a Scl-Ab treatment and in mice carrying the *G170V* HBM mutation did increase all osteoblast parameters (OS/BS, Ob.S/BS, N.Ob/BS) and the bone formation rate (BFR/BS) more pronounced in *Tgif1*^*−/−*^ mice than in control animals (Fig. 4b and Supplementary Tables 5 and 6). Although the exact reason for this observation remains to be elucidated, it could be due to a higher responsiveness on the basis of a low bone turnover remodeling in *Tgif1*^*−/−*^ mice. However, this effect on osteoblasts did not yield a greater increase in bone mass. Thus, these data confirm that Tgif1 is dispensable to elicit the Wnt-induced increase in bone mass.

### PTH induces Tgif1 expression through AP1 signaling

**Fig. 5:**
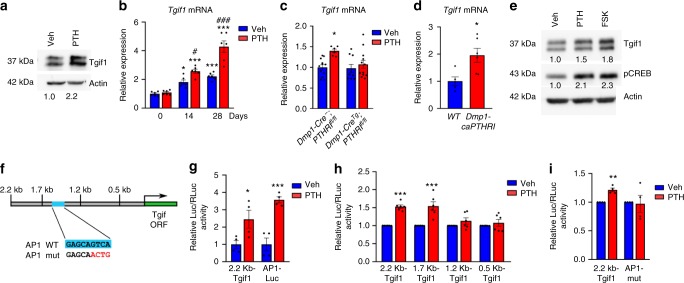
Parathyroid hormone (PTH) induces TG-interacting factor 1 (Tgif1) expression through activator protein 1 (AP1). **a** Immunoblot of Tgif1 expression in osteoblasts upon PTH or vehicle (Veh) treatment (representative image of 4 experiments). **b** Tgif1 expression in differentiating SW3 cells upon PTH or Veh treatment (*N* = 6, 6). **c** *Tgif1* expression in tibiae of *Dmp1-Cre*^*Tg*^*;PTHR1*^*fl/fl*^ mice compared to *Dmp1-Cre*^*−*^*;PTHR1*^*fl/fl*^ control littermates after Veh or PTH injection (*Dmp1-Cre*^*−*^*;PTHR1*^*fl/fl*^ + Veh, PTH: *N* = 12, 6, *Dmp1-Cre*^*Tg*^*;PTHR1*^*fl/fl*^ + Veh, PTH: *N* = 9, 12). **d** *Tgif1* expression in tibiae of *Dmp1-caPTHR1* mice compared to wild-type (WT) littermates (*N* = 5, 6). **e** Immunoblot of Tgif1 and pCREB in osteoblasts after stimulation with PTH, Forskolin (FSK) or Veh (representative image of 3 experiments). **f** Schematic of Tgif1 reporter gene constructs. An AP1 binding site (AP1 WT) was mutated (AP1 mut). **g** Luciferase activity in osteoblasts transfected with a 2.2 kb Tgif1 reporter construct or an AP1-Luc reporter plasmid as positive control and stimulated with Veh or PTH (2.2 kb Tgif1+Veh, PTH: *N* = 4, 4, AP1-Luc+Veh, PTH: *N* = 4, 4) **h** Luciferase activity in ST2 cells transfected with a 2.2 kb Tgif1 reporter construct or different truncations thereof and stimulated with Veh or PTH (2.2 kb Tgif1+Veh, PTH: *N* = 6, 6, 1.7 kb Tgif1+Veh, PTH: *N* = 6, 6, 1.2 kb Tgif1+Veh, PTH: *N* = 6, 6, 0.5 kb Tgif1+Veh, PTH: *N* = 6, 6). **i** Luciferase activity in ST2 cells transfected with a WT 2.2 kb Tgif1 reporter plasmid or with an inactivated AP1 site (AP1-mut) and stimulated with Veh or PTH (2.2 kb Tgif1+Veh, PTH: *N* = 4, 4, AP1-mut+Veh, PTH: *N* = 4, 4). **a**, **e** Immunoblot for Actin was used as a loading control. Normalized fold expression and molecular weight in kilo Dalton (kDa) are indicated. **b**-**d**, **g**-**i** *p<0.05, **p<0.01, ***p<0.001 vs. 0 Days Veh **b**, Veh **c**, **g**-**i** or WT **d**. **b** #*p*<0.05, ###*p*<0.001 vs. 0 Days PTH. Error bars represent the s.e.m. Two-tailed Student's *t*-test was used to compare two groups **c**, **d**, **g**–**i**, and analysis of variance (ANOVA) followed by Newman–Keuls post-hoc analysis was used to compare more than two groups **b**

Upon binding of PTH to its G protein-coupled receptor PTHR1, the Gsα-linked cAMP-dependent protein kinase A (PKA) signaling pathway becomes activated^45,46^. Activated PKA induces phosphorylation of the cAMP response element-binding protein (CREB) in the cytoplasm, which translocates to the nucleus and initiates the expression of target genes^45,46^. Members of the activator protein 1 (AP1) family are increased in their expression upon PTH stimulation and participate in regulating osteoblast function and bone formation^47^. Osteoblast activation by PTH not only increases bone formation but also the expression of RANKL, which activates osteoclast-mediated bone resorption and causes a high turnover bone remodeling^2^. Since the histomorphometric analysis of *Tgif1*^*−/−*^, *Osx-Cre*^*Tg*^*;Tgif1*^*fl/fl*^ and *Dmp1-Cre*^*Tg*^*;Tgif1*^*fl/fl*^ mice revealed a low-turnover bone phenotype (Fig. 1f–i and Supplementary Fig. 2c, d and Supplementary Tables 1–4), we hypothesized that Tgif1 might be a component of the PTH signaling cascade. To investigate this hypothesis, we first determined if *Tgif1* is a PTH target gene. PTH stimulation increased the Tgif1 protein abundance in calvarial osteoblasts and the *Tgif1* mRNA expression in differentiating cells of the osteocyte-derived SW3 cell line (Fig. 5a, b). Furthermore, *Tgif1* expression in bones of mice lacking the PTHR1 in mature osteoblasts and osteocytes (*Dmp1-Cre*^*Tg*^*;PTHR1*^*fl/fl*^)^48^ was unchanged under basal conditions, while PTH increased *Tgif1* expression in control but not in *Dmp1-Cre*^*Tg*^*;PTHR1*^*fl/fl*^ animals (Fig. 5c). In a reciprocal experiment we determined that the constitutive activation of the PTHR1 in mature osteoblasts and osteocytes (*Dmp1-caPTHR1*)^49^ increased the *Tgif1* expression (Fig. 5d) as did the stimulation of calvarial osteoblasts with the PKA activator forskolin (Fig. 5e), demonstrating that Tgif1 expression is induced by the PTH–PKA–pCREB pathway.

To further elucidate the mechanisms by which PTH induces Tgif1 expression, we analyzed the 2.2 kb region of the *Tgif1* promoter. This analysis revealed the presence of an AP1 binding site upstream of the *Tgif1* transcription start site (Fig. 5f), suggesting that PTH might increase Tgif1 expression via AP1 signaling. Indeed, PTH increased the activity of the 2.2 kb fragment of the *Tgif1* promoter in osteoblasts (Fig. 5g). Progressive truncation of the *Tgif1* promoter eliminating the AP1 binding site (Fig. 5h) and its specific mutation (Fig. 5f, i) abolished the reporter gene activation, demonstrating that PTH induces Tgif1 through PKA–pCREB–AP1 signaling.

### PTH increases bone mass in a Tgif1-dependent manner

Since *Tgif1* is a PTH target gene downstream of the PKA–pCREB–AP1 signaling cascade, we proposed that Tgif1 might be involved in the increase in bone mass in response to PTH. To explore this hypothesis, we injected 8-week-old *Tgif1*^*−/−*^ mice and control littermates with PTH or vehicle intermittently for 4 weeks. In control animals, PTH strongly increased all osteoblast parameters (OS/BS, Ob.S/BS and N.Ob/BS) and bone formation (MS/BS, BFR/BS and MAR) as well as osteoclast parameters (Oc.S/BS and N.Oc/BS) and bone resorption (ES/BS), leading to a high bone mass (BV/TV) phenotype (Supplementary Fig. 4a, b and Supplementary Table 8). In *Tgif1*^*−/−*^ mice, PTH had a mild effect on osteoblast parameters and bone formation (OS/BS, Ob.S/BS, N.Ob/BS, BFR/BS and MAR), while the frequency of mineralizing surfaces per bone surface (MS/BS) remained unchanged. PTH also increased the number of osteoclasts per bone surface (N.Oc/BS) as well as the frequency of eroded surfaces per bone surface (ES/BS) and therefore bone resorption in *Tgif1*^*−/−*^ mice. However, these effects were moderate compared to control littermates and, most importantly, did not yield an increase in bone mass (BV/TV) (Supplementary Fig. 4a, b and Supplementary Table 8).

**Fig. 6:**
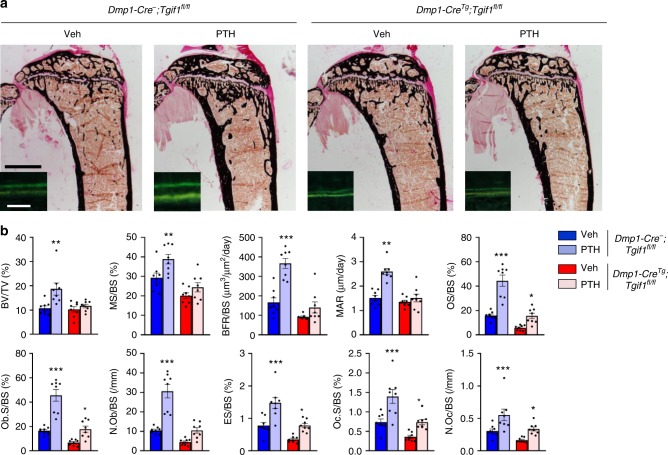
Parathyroid hormone (PTH) elicits its bone anabolic effect in a TG-interacting factor 1 (Tgif1)-dependent manner. **a** Representative images of proximal tibiae of 12-week-old male mice with the genotypes *Dmp1-Cre*^*−*^*;Tgif1*^fl/fl^ and *Dmp1-Cre*^*Tg*^*;Tgif1*^fl/fl^ after von Kossa staining and fluorescence double labeling to visualize bone formation (insets). *Dmp1-Cre*^*−*^*;Tgif1*^fl/fl^ and *Dmp1-Cre*^*Tg*^*;Tgif1*^fl/fl^ mice were treated with PTH or vehicle (Veh) for 4 weeks. Scale bars indicate 1 mm (black) and 50 μm (white). **b** Histomorphometric analysis of the proximal tibiae of 12-week-old *Dmp1-Cre*^*−*^*;Tgif1*^fl/fl^ and *Dmp1-Cre*^*Tg*^*;Tgif1*^fl/fl^ male mice after PTH or Veh treatment for 4 weeks (*Dmp1-Cre*^*−*^*;Tgif1*^fl/fl^+Veh: *N* = 8, *Dmp1-Cre*^*−*^*;Tgif1*^fl/fl^+PTH: *N* = 8, *Dmp1-Cre*^*Tg*^*;Tgif1*^fl/fl^+Veh: *N* = 8, *Dmp1-Cre*^*Tg*^*;Tgif1*^fl/fl^+PTH: *N* = 8). For abbreviations see the legend to Fig. 1. *p<0.05, **p<0.01, ***p<0.001 vs. Veh of the same genotype. Error bars represent the s.e.m. Statistical analysis was performed using analysis of variance (ANOVA) followed by Newman–Keuls post-hoc test

To test whether PTH increases bone mass in mice bearing a *Tgif1* deletion in the osteoblast lineage, we injected PTH for 4 weeks into 8-week-old *Dmp1-Cre*^*Tg*^*;Tgif1*^*fl/fl*^ mice and control littermates. Again, in control animals PTH strongly increased all osteoblast (OS/BS, Ob.S/BS and N.Ob/BS) and osteoclast parameters (Oc.S/BS and N.Oc/BS) as well as bone formation (MS/BS, BFR/BS and MAR) and bone resorption (ES/BS) in the context of a highly activated bone remodeling with the consequence of a significant increase in bone mass (BV/TV) (Fig. 6a, b and Supplementary Table 9). In *Dmp1-Cre*^*Tg*^*;Tgif1*^*fl/fl*^ mice, this effect was attenuated in response to PTH treatment, resulting in a similar phenotype seen in *Tgif1*^*−/−*^ mice. For instance, in contrast to *Tgif1*^*−/−*^ animals the bone formation rate per bone surface (BFR/BS), the mineral apposition rate (MAR) and the number of osteoblasts per bone surface (N.Ob/BS) were not increased in *Dmp1-Cre*^*Tg*^*;Tgif1*^*fl/fl*^ mice in response to PTH. Consistently, bone mass (BV/TV) was also not changed in *Dmp1-Cre*^*Tg*^*;Tgif1*^*fl/fl*^ mice (Fig. 6a, b and Supplementary Table 9). These findings support the notion that Tgif1 is a critical component of the PTH-mediated increase in bone formation and bone mass accrual.

### PTH reduces sclerostin expression in part through Tgif1

**Fig. 7:**
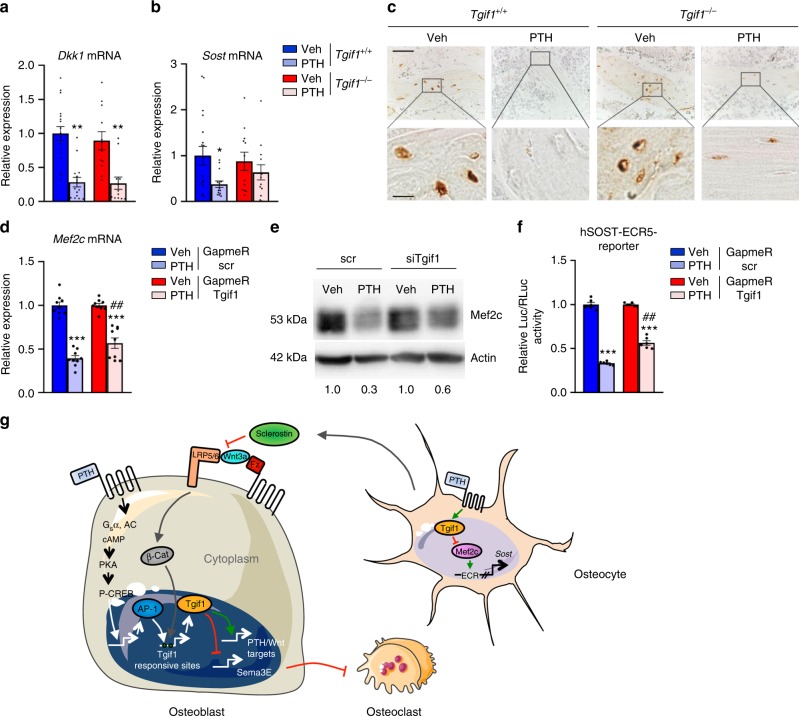
Suppression of sclerostin expression by parathyroid hormone (PTH) depends in part on TG-interacting factor 1 (Tgif1). **a** Relative expression of *Dkk1* mRNA and of **b** *Sost* mRNA in tibiae of *Tgif1*^*+/+*^ and *Tgif1*^*−/−*^ mice 4 h after injection of PTH or vehicle (Veh) (*Tgif1*^*+/+*^+Veh: *N* = 18, *Tgif1*^*+/+*^+PTH: *N* = 14, *Tgif1*^*−/−*^+Veh: *N* = 12, *Tgif1*^*−/−*^+PTH: *N* = 13). **c** Immunohistochemical staining of sclerostin expression in tibiae of *Tgif1*^*+/+*^ and *Tgif1*^*−/−*^ mice after treatment with PTH or Veh. Scale bars indicate 50 μm (lower magnification, upper panel) and 10 μm (higher magnification, lower panel). **d** Relative expression of *Mef2c* mRNA in UMR-106 cells. Cells were transfected with scrambled (scr) control GapmeR or GapmeR targeting Tgif1. After 48 h, cells were stimulated with PTH or Veh for 8 h (*N* = 3). **e** Immunoblot demonstrating the protein abundance of Mef2c in UMR-106 cells. Cells were transfected with scr siRNA or siRNA targeting Tgif1 (siTgif1). After 48 h, cells were stimulated with PTH or Veh for 8 h. Immunoblot for Actin was used as a loading control. Normalized fold expression and molecular weight in kilo Dalton (kDa) are indicated (representative image of 3 experiments). **f** UMR-106 cells were transfected with scr GapmeR or GapmeR targeting Tgif1 and 24 h later with a hSOST-ECR5 reporter construct. After 8 h, cells were stimulated with PTH or Veh for 16 h. Shown is the relative luciferase activity (GapmeR scr+Veh: *N* = 3, GapmeR scr+PTH: *N* = 3, GapmeR Tgif1+Veh: *N* = 3, GapmeR Tgif1+PTH: *N* = 3). **a**, **b** *p<0.05, **p<0.01 vs. Veh of the same genotype. **d**, **f** ***p<0.001 vs. the respective GapmeR+Veh, ^##^*p*<0.005 vs. GapmeR scr+PTH. Error bars represent the s.e.m. Statistical analysis was performed using analysis of variance (ANOVA) followed by Newman–Keuls post-hoc test. **g** Schematic drawing of the function of Tgif1 in osteoblasts, osteocytes and in response to the activation of the canonical Wnt and PTH bone anabolic pathways

The bone anabolic effect of PTH is in part mediated through cross-activation of other pathways including canonical Wnt signaling^31,32,46,50,51^. For instance, stimulation of osteoblasts and osteocytes with PTH decreases the expression of the canonical Wnt inhibitors Dkk1 and sclerostin, the product of the *SOST* gene^30,31,52,53^. Since the Wnt pathway is a strong inducer of bone formation^15^, antagonizing inhibitors of this pathway leads to an increase in bone mass. To investigate whether Tgif1 is implicated in the PTH-mediated cross-activation of the Wnt pathway, we quantified the expression of Dkk1 and sclerostin in the long bones of *Tgif1*^*−/−*^ mice and control littermates 4 h after injection of PTH or vehicle. Gene expression analysis revealed that PTH fully reduced the expression of *Dkk1* in bones of *Tgif1*^*−/−*^ mice and control littermates (Fig. 7a), while the expression of *Sost* mRNA was only partially and not statistically significantly reduced in mice lacking Tgif1 (Fig. 7b). To confirm this observation, we performed immunohistochemistry to detect the abundance of sclerostin in the bones of mice treated with PTH. Consistent with the results of the gene expression analysis, sclerostin protein expression in osteocytes was fully abrogated by PTH in control animals and only partially reduced in *Tgif1*^*−/−*^ mice (Fig. 7c). These findings demonstrate that Tgif1 in mature osteoblasts and osteocytes is necessary for the full PTH-mediated inhibition of sclerostin expression.

To unravel the molecular mechanism by which Tgif1 contributes to the suppression of sclerostin expression, we analyzed the 8 kb upstream region of the *Sost* promoter and identified 11 putative sites to which Tgif1 could bind (Supplementary Fig. 5a). Tgif1 forms complexes with other factors and represses gene transcription^54^. We therefore hypothesized that PTH might increase the *Sost* promoter occupancy by Tgif1 and attenuate its activity. To test this hypothesis, we performed chromatin immunoprecipitation (ChIP) of Tgif1 in the OCY454 osteocyte-like cell line 2 h after incubation with PTH or vehicle. The ChIP assays revealed that Tgif1 associates with all sites investigated, except binding sites 8 and 10 (Supplementary Fig. 5b). Binding to site 9 was detectable but did not reach significance (Supplementary Fig. 5b). In contrast to our hypothesis, treatment with PTH caused a dissociation of Tgif1 from all sites with the exception of sites 7 and 9 where no significance was reached (Supplementary Fig. 5b). As a positive control, binding of Tgif1 to the promoter of *retinoic acid receptor alpha* (*Rar alpha*) and its dissociation upon PTH treatment was confirmed (Supplementary Fig. 5b)^55^. These observations suggest that Tgif1 might not have a suppressive effect on the *Sost* promoter activity in response to PTH treatment and that other mechanisms might exist.

In osteocytes, Mef2 transcription factors activate a distant enhancer of the *SOST* gene corresponding to the region that is deleted in Van Buchem disease^32^. Furthermore, PTH-mediated inhibition of sclerostin expression in response to PTH stimulation is largely due to a decrease in Mef2c expression^32^. Interestingly, while the distal enhancer of the *SOST* gene does not contain Tgif1 consensus sequences, the *Mef2c* promoter contains several Tgif1 binding sites. This indicates that Tgif1 might contribute to the control of sclerostin expression in an indirect manner by participating in the repression of Mef2c transcription in response to PTH treatment. Indeed, PTH suppressed *Mef2c* mRNA expression and protein abundance to a much lesser extent in Tgif1-deficient cells (Supplementary Fig. 6a, b) compared to controls (Fig. 7d, e). To determine the functional relevance of this observation, we analyzed the activity of a gene reporter construct containing the Mef2-responsive distal enhancer element of the human *SOST* gene^32^. Consistent with our findings, PTH strongly suppressed the reporter gene activity in control cells but to a significantly lower degree in cells in which the expression of Tgif1 had been restricted (Fig. 7f), demonstrating the functional role of Tgif1 in regulating the *SOST* gene activity.

In summary, in response to PTH treatment, Tgif1 dissociates from the *SOST* promoter and inhibits the expression of Mef2c. Due to a reduced abundance of Mef2c, a distant enhancer element of the *SOST* gene containing Mef2c binding sites becomes less activated, causing a decrease in sclerostin expression and an increase in bone formation. In the absence of Tgif1, this regulatory cascade is disturbed, leading to an impaired decrease of sclerostin expression in response to PTH treatment and a lower bone formation. Thus, Tgif1 is an important mediator of the gain in bone mass in response to PTH treatment.

## Discussion

In this study, we identified Tgif1 as a novel stimulator of osteoblast differentiation and function. Bone remodeling depends on Tgif1, since absence of Tgif1 in osteoblasts causes low bone turnover due to a decreased osteoblast function and bone formation. The impaired bone formation is accompanied by an osteoblast-mediated decrease in osteoclast activity and bone resorption. Since the amount of bone resorbed equals the amount of bone formed, bone mass is unchanged in Tgif1-deficient mice.

Interestingly, osteoclast differentiation and function is reduced upon deletion of Tgif1 in osteoblasts. After changes in the RANKL/OPG system, in Ephrin signaling and in the expression of interleukins and insulin-like growth factors were excluded, Sema3E was found to be highly expressed by *Tgif1*^*−/−*^ osteoblasts and to mediate the suppression of osteoclast differentiation. Although an osteoblast-targeted deletion of *Sema3E* is needed to determine whether this alleviates the reduced bone resorption in mice lacking Tgif1 in osteoblasts, our findings underscore the important function of Sema3E in bone cell interaction.

In breast cancer, Tgif1 expression correlated with a poor prognosis and supported the Wnt1-driven cancer development^42^. In colorectal cancer, Tgif1 is indispensable for canonical Wnt activation and promotes the disease through Wnt activation^43^. The role of Tgif1 in oncology let us to speculate whether Tgif1 might also be implicated in Wnt signaling in bone. Consistent with the findings in cancer cells, Wnt signaling increased Tgif1 expression in osteoblasts. This increase was mediated by β-catenin-Tcf/Lef signaling since eliminating Tcf/Lef binding sites in the *Tgif1* promoter abolished the transcriptional activation. In contrast to cancer cells, absence of Tgif1 in bone or osteoblastic cells did not change the basal activity of the Wnt pathway in vitro or in vivo. We further determined that Tgif1 is dispensable for Wnt-mediated increase in bone mass. This discrepancy to cancer might be due to differences in the molecular regulation of Wnt signaling pathways under malignant versus benign conditions and suggests that Wnt signaling might function differently in bone than in other organs.

PTH binds to the PTH/PTHrP receptor (PTHR1), a class II G protein-coupled receptor that activates several pathways, including the Gq/11-linked phosphatidyl inositol-specific phospholipase C–protein kinase C signaling pathway and the Gsα-linked cAMP-dependent protein kinase A (PKA) signaling pathway^45,46^. Our in vitro and in vivo data demonstrate that Tgif1 expression in osteoblasts and osteocytes is downstream of the PTHR1. Further analysis revealed that Tgif1 expression is induced by PKA–CREB signaling. Downstream of PTHR1, PKA signaling leads to the phosphorylation of CREB, which induces the expression of AP1 transcription factors^45–47^. AP1 signaling is a mediator of the anabolic effect of PTH^47^. The functional relevance of an AP1 binding site in the *Tgif1* promoter was demonstrated using truncations and a specific mutation in reporter assays. These experiments establish *Tgif1* as a PTH target gene downstream of the PTHR1–PKA–pCREB–AP1 cascade.

Similar to the Wnt pathway, Tgif1 expression is increased by PTH treatment. Although Tgif1 is dispensable for the bone anabolic function of Wnt signaling, we addressed the question of whether Tgif1 might be implicated in the increase in bone mass by PTH treatment. This hypothesis was supported by a low-turnover bone remodeling in the absence of Tgif1, which represents a somehow opposite phenotype obtained by intermittent administration of PTH. In a pharmacological context, PTH augments osteoblast function and bone formation but also the production of RANKL, thereby activating osteoclasts and bone resorption^2^. This causes a high turnover bone remodeling with a net gain in bone mass. Indeed, daily injections of PTH into mice with a germline deletion of *Tgif1* or a targeted deletion of *Tgif1* in osteoblasts and osteocytes prevented the increase in bone mass compared to control littermates. However, absence of Tgif1 did not fully abrogate all effects of PTH on bone remodeling and some differences were noticed between the two mouse models of *Tgif1* deletion. These findings indicate that Tgif1 contributes to many but not all effects downstream of PTHR1 signaling. Most importantly, in the absence of Tgif1 in the osteoblast lineage, the residual effects of PTH on bone are insufficient for bone mass accrual.

PTH exerts its bone-forming function also in part through cross-activation of canonical Wnt signaling^46^. For instance, PTH inactivates glycogen synthase kinase-3β (GSK3β)^50^ and stabilizes β-catenin^51^. PTHR1 forms a complex with Lrp6 upon binding of PTH, thereby disintegrating the destruction complex^51^. In this context, Lrp6 in osteoblasts was shown to be essential for the anabolic effect of PTH^51^. In osteocytes, PTH reduces the expression of the Wnt inhibitors Dkk1^30^ and sclerostin^49^. Inhibition of sclerostin expression in osteocytes is mediated by Mef2 transcription factors that control the activity of a distant enhancer element of the *SOST* gene^32^. In detail, PTH signaling leads to a PKA-mediated phosphorylation and inhibition of the salt-inducible kinase 2 (SIK2). This reduces the phosphorylation of histone deacetylases 4 and 5 (HDAC4/5), which translocate to the nucleus and inhibit the Mef2c-driven sclerostin expression^56^. Evidence for the physiological role of sclerostin inhibition as an important component of the PTH anabolic effect is provided by in vivo studies, demonstrating that PTH decreases sclerostin expression in wild-type mice. Furthermore, the PTH-induced gain in bone mass was blunted in mice overexpressing or lacking sclerostin, demonstrating that sclerostin is an important component of anabolic PTH signaling^57^. However, other studies have shown a normal or even enhanced response to PTH treatment in *SOST* knockout mice^46^. Consistent with these findings, the increase in bone mass by PTH in mice overexpressing human sclerostin under the control of the 8 kb fragment of the *Dmp1* promoter was comparable to control littermates^58^. These controversies might be explainable by the use of transgenes obtained from different species, the use of various artificial promoter elements to drive the overexpression or other technical aspects. Nevertheless, although the exact contribution of the downregulation of sclerostin expression in response to PTH is not yet fully elucidated, this mechanism is likely to be an important component of the PTH bone anabolic effect.

In order to determine whether the PTH-mediated crosstalk with the Wnt pathway could be impaired in *Tgif1*^*−/−*^ mice in which PTH treatment did not increase bone mass, we investigated the Dkk1 and sclerostin expression in bones from *Tgif1*^*−/−*^ mice and control littermates. The basal expression of *Dkk1* and *Sost* was comparable between *Tgif1*^*−/−*^ and control mice. Of note, PTH treatment suppressed the *Dkk1* expression in animals of both genotypes strongly and equally but the *Sost* expression was only partially decreased in *Tgif1*^*−/−*^ mice compared to control littermates. This observation was confirmed in bone sections at the protein level using immunohistochemistry. Although the incomplete downregulation of sclerostin expression by PTH in *Tgif1*^*−/−*^ mice was moderate, it might at least in part contribute to the blunted bone anabolic effect of PTH in Tgif1-deficient mice.

Although Tgif1 physically associates with the *Sost* promoter, an inhibitory effect on sclerostin expression is less likely due to the displacement of Tgif1 from its consensus binding sites in response to PTH stimulation. However, our findings demonstrate that Tgif1 contributes to the PTH-mediated repression of Mef2c expression. By this mechanism, Tgif1 has an indirect inhibitory effect on sclerostin expression. In the absence of Tgif1, PTH stimulation reduces Mef2c expression incompletely, allowing the remaining Mef2c to bind to a distant enhancer element of the *Sost* gene, leading to a residual transcriptional activity and sclerostin expression.

In summary, the PTH–PKA–pCREB–AP1 pathway and canonical Wnt signaling increase Tgif1 expression in osteoblasts and osteocytes (Fig. 7g). Tgif1 supports osteoblast differentiation, function and bone formation. By suppressing the expression of Sema3E, Tgif1 facilitates bone resorption and therefore physiological bone remodeling. In osteocytes, Tgif1 contributes to the inhibition of Mef2c expression and thereby to the decrease of sclerostin expression in response to PTH treatment. Thus, our study identified Tgif1 as a novel regulator of bone remodeling and an essential component of the PTH anabolic action.

## Methods

### Mouse models

Mice with a germline deletion of *Tgif1* and a loxP-flanked *Tgif1* gene have been reported previously^34^. To delete *Tgif1* in osteoblasts in vivo, mice expressing the Cre recombinase under the control of the *Osterix* (*Osx/SP7*) promoter (*Osx-Cre*^*Tg*^)^36^ or an 8 kb fragment of the murine *Dentin matrix protein 1* (*Dmp1*) (*Dmp1-Cre*^*Tg*^)^37^ promoter were crossed with mice in which exons 2 and 3 of the *Tgif1* gene are flanked by loxP sites (*Tgif1*^*fl/+*^)^34^. The resulting mice of the genotype *Osx-Cre*^*Tg*^*;Tgif1*^*fl/+*^ were mated with *Tgif1*^*fl/+*^ mice to obtain *Osx-Cre*^*Tg*^*;Tgif1*^*fl/fl*^ mice with a conditional deletion of *Tgif1* in osteoblasts. *Osx-Cre*^*Tg*^*;Tgif1*^*+/+*^ mice were used as control to exclude a non-specific phenotype caused by the Cre expression. Since no bone phenotype was observed in *Dmp1-Cre*^*+*^ mice, *Dmp1-Cre*^*-*^*;Tgif1*^*fl/fl*^ mice were used as controls. Mice carrying the *Lrp5*^*G170V/+*^ and *Lrp5*^*A213V/+*^ HBM mutations were obtained from Jackson Laboratories (Bar Harbor). Mice with a conditional ablation of the *PTHR1* controlled by a 10 kb fragment of the *Dmp1* promoter (*Dmp1-Cre*^*Tg*^*;PTHR1*^*fl/fl*^) and mice expressing a constitutively active PTHR1 (caPTHR1) regulated by an 8 kb fragment of the *Dmp1* promoter (*Dmp1-caPTHR1*) have been described previously^48,49^. For bone anabolic studies, a recombinant fragment of human parathyroid hormone (PTH 1–34; 100 µg/kg of body weight, Biochem) was administered intraperitoneally 5 times a week for 4 weeks. Anti-sclerostin antibody (Scl-Ab; kindly provided by Novartis and Mereo BioPharma) was delivered intravenously at a concentration of 100 µg/kg once a week for 4 weeks. All mouse strains used were maintained on a C57Bl/6J background. Sample sizes were determined according to the standards used in the field. All experimental animals that were alive by the time of analysis were included in the study. Mice of the same genotype were randomized to the treatment or control group. Investigators were not blinded to the group allocation during the experiment, but assessment of the outcome by bone histomorphometry was performed in a blinded manner. The study received approval by the local authority for animal welfare and experiments were conducted in compliance with all relevant ethical regulations for animal testing and research.

### Bone analyses

Mice were injected 7 and 2 days before sacrifice with calcein (20 mg/kg) and demeclocycline (20 mg/kg; both Sigma-Aldrich), respectively. Tibiae and the fourth lumbar vertebral bodies (L4) were collected and fixed in 3.7% phosphate-buffered saline (PBS)-buffered formaldehyde. For histomorphometric analysis, tibiae and L4 were embedded in methylmethacrylate. Toluidine blue, von Kossa and TRAP staining were performed using 4 µm sagittal sections. Quantitative bone histomorphometric measurements were performed according to standard protocols^59^ using an OsteoMeasure system (OsteoMetrics). Microcomputed tomography (µCT) was used for bone analyses. Distal and midshaft femora were analyzed using high-resolution µCT with a fixed isotropic voxel size of 15.6 µm (70 kV at 114 μA, 400 ms integration time; Viva80 micro-CT; Scanco Medical AG). All analyses were performed on digitally extracted bone tissue using three-dimensional distance techniques^60^.

### Cell culture and ex vivo osteoblast differentiation assays

BMSCs were obtained from three different mice. For each cell preparation, cells were plated at a density of 10,000 cells/cm^2^ in 3 parallel cultures and cultured for 2 days in α-minimum essential medium (α-MEM; Life Technologies) containing 10% fetal bovine serum (FBS; Life Technologies) and 100 U/ml penicillin; 100 µg/ml streptomycin (P/S; Life Technologies). Prior to RNA isolation, BMSCs from 3 parallel cultures were pooled. The mouse stromal cell line ST2 was purchased from DSMZ (Cat. No.: ACC 333) and maintained in RPMI-1640 medium (Life Technologies) supplemented with 10% FBS and P/S. The rat osteosarcoma cell line UMR-106 was purchased from ATCC (Cat. No.: CRL-1661) and maintained in Dulbecco's modified Eagle's medium (DMEM; Life Technologies) with 10% FBS and P/S. The osteocyte-derived OCY454 cell line has been reported previously^61^ and the osteocyte-derived IDG-SW3 cell line was kindly provided by Dr. Lynda Bonewald. Cells were maintained and expanded in culture dishes coated with rat tail type I collagen (Corning) at 33 °C in complete medium (α-MEM, 10% FBS, and P/S) in the presence of 10 U/ml interferon-gamma. Differentiation was performed at 37 °C in the absence of interferon-gamma but with the addition of 0.2 mM L-ascorbic acid (Sigma) and 5 mM β-glycerophosphate (Millipore). For calvarial osteoblast cultures, calvariae were dissected from 1- to 3-day-old mice and digested sequentially in α-MEM containing 0.1% collagenase and 0.2% dispase (both Roche). Cell fractions 2 to 4 were combined and expanded in α-MEM containing 10% FBS and P/S. Osteoblast differentiation was induced by supplementing α-MEM with 50 µg/ml L-ascorbic acid and 5 mM β-glycerophosphate. Osteoblast differentiation was determined by alkaline phosphatase (ALP) and Alizarin Red staining after fixing the cells in 4% neutrally buffered formaldehyde solution. For ALP staining, cells were incubated with naphthol ASMX/Fast Blue (both from Sigma-Aldrich) in Tris-HCl solution at pH 8.4 for 15 min at room temperature. To detect matrix mineralization, Alizarin Red staining was performed with 40 mM Alizarin Red S (AR-S; Sigma-Aldrich) solution at pH 4.2 for 10 min at room temperature.

Long bone osteoblasts were isolated from femora and tibiae of 8–10-week-old mice. After removing muscles in sterile PBS, bone marrow was flushed and bones were cut in small pieces. Bone pieces were digested with 0.1% collagenase for 2 h at 37 °C and plated in α-MEM containing 10% FBS and P/S. Outgrowing osteoblasts were detached with trypsin after 1 week and cultured until confluence (2–3 weeks). Bone marrow macrophages (BMMs) were isolated from the bone marrow of 8-week-old C57Bl/6J mice. Non-adherent cells were collected after 3 h of incubation on plastic and cultured in α-MEM containing 10% FBS, P/S and macrophage colony stimulating factor (100 ng/ml, PeproTech). For osteoblast–osteoclast co-cultures, long bone osteoblasts were plated on 96-well plates and stimulated with Vitamin D and Prostaglandin E2 (PGE2). One day later, BMMs were plated over osteoblasts. Cultures were terminated after 5 days, fixed and stained with TRAP for 10 min at 37 °C. All reagents of the TRAP solution (Naphthol-ASMX-Phosphate, Fast Red Violet LB-Salt and *N*,*N*-Dimethylformamid) were purchased from Sigma. No cell line used is listed in the database of commonly misidentified cell lines maintained by ICLAC. Cell lines used in this study have not been authenticated but have been regularly tested for mycoplasma contamination using the PCR Mycoplasma Test Kit (PromoKine).

### Transfection of siRNAs and GapmeRs

Scrambled siRNAs and siRNAs targeting mouse Sema3E and rat Tgif1 were purchased from Dharmacon. Scrambled GapmeRs and GapmeRs targeting rat Tgif1 were purchased from Qiagen. Long bone osteoblasts were transfected using the NEON Transfection system (Thermo Fisher Scientific). UMR-106 rat osteosarcoma cells were transfected using Lipofectamine 3000 (Life Technologies).

### DNA constructs and Luciferase assays

To mutate the *Tgif1* promoter reporter construct (kindly provided by S.J. Brandt), the AP1 binding site was modified using the QuickChange II XL site-directed mutagenesis kit (Agilent). ST2 cells were transfected with progressively truncated *Tgif1* reporter constructs and a reporter construct bearing a mutated AP1 binding site along with a *Renilla* reporter using Lipofectamine reagent (Life Technologies) according to the supplier’s recommendations. TopFlash β-catenin luciferase reporter (Addgene) containing 7 concatenated Tcf/Lef binding sites were co-transfected with a plasmid encoding β-catenin as well as a *Renilla* luciferase reporter plasmid in calvarial osteoblasts using the NEON transfection system. UMR-106 cells were transfected with the hSOST-ECR5-luc reporter construct^32^ and a *Renilla* luciferase reporter plasmid using Lipofectamine 3000. Luciferase assays were performed using the Dual-Glo Luciferase Reporter Gene Assay System (Promega) according to the instructions provided by the manufacturer. Firefly luciferase activity was normalized to *Renilla* luciferase activity.

### Gene expression analyses

Total RNA was isolated from mouse bones using TRIzol reagent (Life Technologies) and from cultured cells using the RNeasy Plus Mini kit (Qiagen) according to the manufacturer’s instructions. Complementary DNA (cDNA) was synthesized from 1 µg of total RNA using the ProtoScript First Strand cDNA Synthesis Kit (NEBioLabs). Quantitative real-time PCR (qRT-PCR) was performed using SYBR Green (Bio-Rad). After normalization to TATA-binding protein (Tbp) mRNA, relative expression levels and fold induction of each target gene were calculated using the comparative CT (ΔΔCT) method. All oligonucleotides used for qPCR analysis are listed in Supplementary Table 10.

### RNA sequencing

Next-generation RNA sequencing was performed using three distinct mouse bone marrow-derived mesenchymal stromal cell populations. Each cell preparation was plated in three cultures and combined prior to analysis. Oligo dT purified mRNA was used to generate cDNAs that were indexed using the standard TruSeq Kits 12-Set A and 12-Set B on Illumina RTA version 1.17.21.3. Read mapping was performed using a robust pipeline involving MAPRSeq v.1.2.1, TopHat 2.0.6 alignment, HTSeq gene counting and expression normalization using EdgeR^62^. Gene expression is expressed in reads per kilobase pair per million mapped reads (RPKM).

### Stable isotope labeling by amino acids in cell culture

Calvarial osteoblasts were isolated from *Tgif1*^*−/−*^ mice and control littermates followed by expansion for 6 days in non-differentiation medium. Cells were re-plated at matching cell numbers for both genotypes not exceeding 80% confluence and cultured overnight. SILAC was performed for 12 h in DMEM (Gibco) supplemented with 0.1% dialyzed FBS lacking Met, Lys and Arg. Amino acid re-supplementation was achieved using l-Lys and l-Arg (Gibco) for control osteoblasts and 13C6 l-Lys and 13C6 l-Arg (Thermo Scientific) for *Tgif1*^*−/−*^ osteoblasts. Met was replaced by L-Azidohomoalanin (AnaSpec) to allow for Click-iT-based enrichment. Conditioned medium obtained from *Tgif1*^*−/−*^ and control osteoblasts was combined in a 1:1 ratio. Cell debris was removed by centrifugation and protease inhibitors (Roche) were added to the solution. Next, newly synthesized proteins were enriched from the conditioned medium using ultra centrifugal filters with a 3 kDa cutoff (Amicon) and processed using the Click-iT Protein Enrichment-Kit (Thermo Fisher) according to the manufacturer’s protocol.

### Proteome analysis

For proteome analysis, proteins were subjected to in-solution digestion with trypsin. Briefly, proteins were reduced with 100 mM dithiothreitol dissolved in 100 mM NH_4_HCO_3_ at 50 °C for 10 min, followed by alkylation with 300 mM iodoacetamid dissolved in 100 mM NH_4_HCO_3_ for 30 min at room temperature. Proteins were incubated with trypsin for 16 h at 37 °C. The digests were acidified with formic acid (FA) and evaporated. For analysis of the tryptic peptides with liquid chromatography coupled to mass spectrometry (LC-MS), samples were dissolved in 20 µl 0.1% FA. LC-MS measurements were performed by injecting the samples on a nano liquid chromatography system (Dionex UltiMate 3000 RSLCnano, Thermo Scientific) coupled via electrospray-ionization to a linear trap quadrupole orbitrap mass spectrometer (Orbitrap Fusion, Thermo Scientific)^63^. Peptides were separated by reversed phase chromatography (buffer A: 0.1% FA, buffer B: 99.9% CAN, 0.1% FA; flow-rate: 250 nl/min; gradient: 2–30% B in 90 min). Every second, a MS scan was performed over a *m/z* range from 400 to 1500, with a resolution of 120,000 FWHM (full width at half maximum) at *m/z* 200 (transient length = 256 ms, maximum injection time = 50 ms, automatic gain control (AGC) target = 2e5). Analysis of fragmented peptide ions in the mass spectrometer was carried out in data-dependent acquisition mode, using the top speed mode, a HCD (higher-energy collisional dissociation) collision energy of 35%, an intensity threshold of 5e3 and an isolation width of 1.5 *m/z*. Fragment spectra were recorded in the ion trap (scan-rate = 66 kDa/s, maximum injection time = 70 ms, AGC target = 1e4). LC-MS raw data were processed with Proteome Discoverer 1.4 (Thermo Scientific). For identification, fragment spectra were searched with Sequest HT against a mouse database (SwissProt, www.uniprot.org, downloaded 1.5.2014). The searches were performed using the following parameters: precursor mass tolerance was set to 10 parts per million (ppm) and fragment mass tolerance was set to 0.5 Da. Furthermore, two missed cleavages were allowed and a carbamidomethylation on cysteine residues as a fixed modification. An oxidation of methionine residues and a ^13^C_6_-label on both lysine and arginine residues were allowed as variable modifications. Peptides were identified with a false discovery rate of 1% using percolator and proteins were kept as correctly identified if at least two unique peptides were identified. For SILAC quantitation, event detector and precursor ion quantifier algorithms of Proteome Discoverer were used^64^. A mass variability of 2 ppm and a 0.2 min retention time tolerance on precursor ion pairs were used and protein ratios were based on the median peptide ratio. At least two isotopic peaks were required for inclusion, as well as a minimal signal to noise level of three.

### Immunoblotting

Cells were lysed in low salt mRIPA buffer (pH 7.5) containing 50 mM Tris base, 150 mM NaCl, 0.5% Nonidet P-40, 0.25% sodium deoxycholate and complete protease and phosphatase inhibitors (Roche). Lysates were separated on 12% polyacrylamide gels and subjected to immunoblot blot analysis. Immunoblots were incubated overnight at 4 °C with primary antibodies against Tgif1 (1:1000, rabbit monoclonal, Abcam, Cat. No: ab52955), Tgif2 (1:500, rabbit polyclonal, Millipore, Cat. No: 09-718), Sema3E (1:1000, goat polyclonal, R&D Systems, Cat. No: AF3239), pCREB (1:500, rabbit monoclonal, Cell Signaling, Cat. No: 87G3), Mef2c (1:1000, rabbit monoclonal, Abcam, Cat. No: 197070) and Actin (1:5000, mouse monoclonal, Millipore, Cat. No: MAB1501). Peroxidase-labeled anti-rabbit or anti-mouse secondary antibodies (1:10,000, Promega, Cat. No: W401B, W402B) were used to visualize bands using the Clarity Western ECL Substrate (Bio-Rad). Immunoblot images were acquired using the ChemiDoc imaging system and Image Lab software (Bio-Rad). Unprocessed scans of the most important immunoblots are supplied in the source data file.

### Immunohistochemistry

To determine sclerostin expression in mouse bones, mice were sacrificed 24 h after the last PTH injection and tibiae were fixed as described above. Bones were decalcified in 10% EDTA (pH 7.4) for 2 weeks, dehydrated, embedded in paraffin and cut in 4 µm sections. Staining for sclerostin was performed using an anti-sclerostin antibody (1:50, goat polyclonal, R&D Systems, Cat. No: AF1589).

### ELISA

Enzyme-linked immunosorbent assay (ELISA) was used for quantitative determination of RANKL and OPG (both Immunodiagnostics systems) in mouse serum. All procedures were performed according to the manufacturer’s guidelines.

### Chromatin immunoprecipitation

Putative Tgif binding sites in the *SOST* promoter were identified using the online platform ALGGEN-PROMO. ChIP was performed using the A/G MAGNA ChIP kit (Millipore, Cat. No: 17-10085) according to the manufacturer’s instruction. Briefly, 5 × 10^6^ cells of the osteocyte-like cell line OCY454 were plated into 15 cm^2^ dishes to differentiate for 2 weeks at 37 °C. After 2 h of treatment with either PTH (100 nM) or vehicle, crosslinking was performed using 1% formaldehyde for 10 min, followed by quenching for 5 min with glycin. After chromatin isolation, DNA was sheared into fragments ranging from 100 bp to 500 bp by 30 cycles of high-frequency sonication using a BioraptorPlus. ChIP was performed using 5 μg of rabbit polyclonal anti-Tgif1 antibody (Santa Cruz Biotechnology, Cat. No: sc9084) or 5 µg of a ChIP-grade polyclonal rabbit anti-IgG antibody (Abcam, Cat. No: ab37415). The amount of DNA pulled down was quantified by qPCR. All Ct values exceeding 34 were excluded from the analysis. ChIP-qPCR data were first normalized to the input of each precipitation using the formula (2^(Ct 100% input−Ct sample)^), followed by normalization to the IgG control. Rarα was used as positive control^55^. The oligonucleotides used for amplifying the DNA fragments representing the 11 Tgif1 binding sites are listed in Supplementary Table 10. A region nearby the transcriptional start site of the *Sost* gene was used as negative control.

### Statistical analyses

Quantitative data are presented as mean ± s.e.m. unless otherwise described. Parametric data were analyzed using an appropriate two-tailed Student's *t*-test when two groups were compared. A one-way analysis of variance (ANOVA) was used when more than two groups were compared, followed by Newman–Keuls post-hoc analysis to compare the groups. Probability values were considered statistically significant at *p* < 0.05. Experiments were repeated at least three times as biological replicates with minimum of two technical replicates. No sample size calculations were performed but the number of mice analyzed in animal studies was determined in agreement with the standards in the field. Variation between groups was similar in all cases. Statistical analyses were performed using GraphPad Prism software.

### Reporting summary

Further information on experimental design is available in the Nature Research Reporting Summary linked to this article.

## Supplementary information

Supplementary Information

Description of Additional Supplementary Files

Reporting Summary

Source Data

## Data Availability

Next generation RNA-sequencing data that support the findings of this study have been deposited at the Center for Biotechnology Information with the accession code GSE89132. The mass spectrometry proteomics data that support the findings of this study have been deposited to the ProteomeXchange Consortium via the PRIDE partner repository with the dataset identifier PXD012303.
