# Supplementary material for: TG-interacting factor 1 (Tgif1)-deficiency attenuates bone remodeling and blunts the anabolic response to parathyroid hormone
Source: Nat Commun. 2019 Mar 22;10:1354. doi: 10.1038/s41467-019-08778-x (PMC6430773; doi:10.1038/s41467-019-08778-x)
Supplement: Supplementary file 1 — Supplementary Information [file 41467_2019_8778_MOESM1_ESM.pdf]

Supplementary information

TG-interacting factor 1 (Tgif1)-deficiency attenuates bone remodeling and blunts the anabolic response to parathyroid hormone

Saito et al.

Supplementary Figure 1

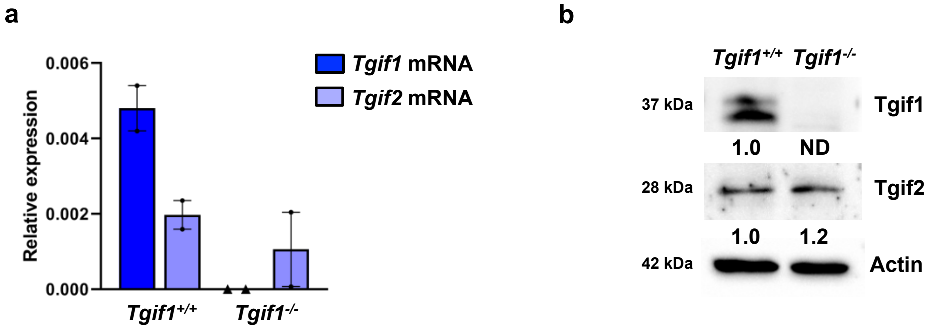

Supplementary Fig. 1 TG-interacting factor 2 (Tgif2) expression is unchanged in the absence of TG-interacting factor 1 (Tgif1). **a** Relative expression of *Tgif1* and *Tgif2* mRNA in calvarial osteoblasts obtained from *Tgif1*$^{+/+}$ and *Tgif1*$^{-/-}$ mice. (*Tgif1*$^{+/+}$ N = 2, *Tgif1*$^{-/-}$ N = 2). **b** Immunoblot of Tgif1 and Tgif2 protein expression in calvarial osteoblasts obtained from *Tgif1*$^{+/+}$ and *Tgif1*$^{-/-}$ mice. Immunoblot for Actin was used as a loading control. Normalized fold expression and molecular weight in kilo Dalton (kDa) are indicated (representative image of 2 experiments), ND = non-detectable

Supplementary Figure 2

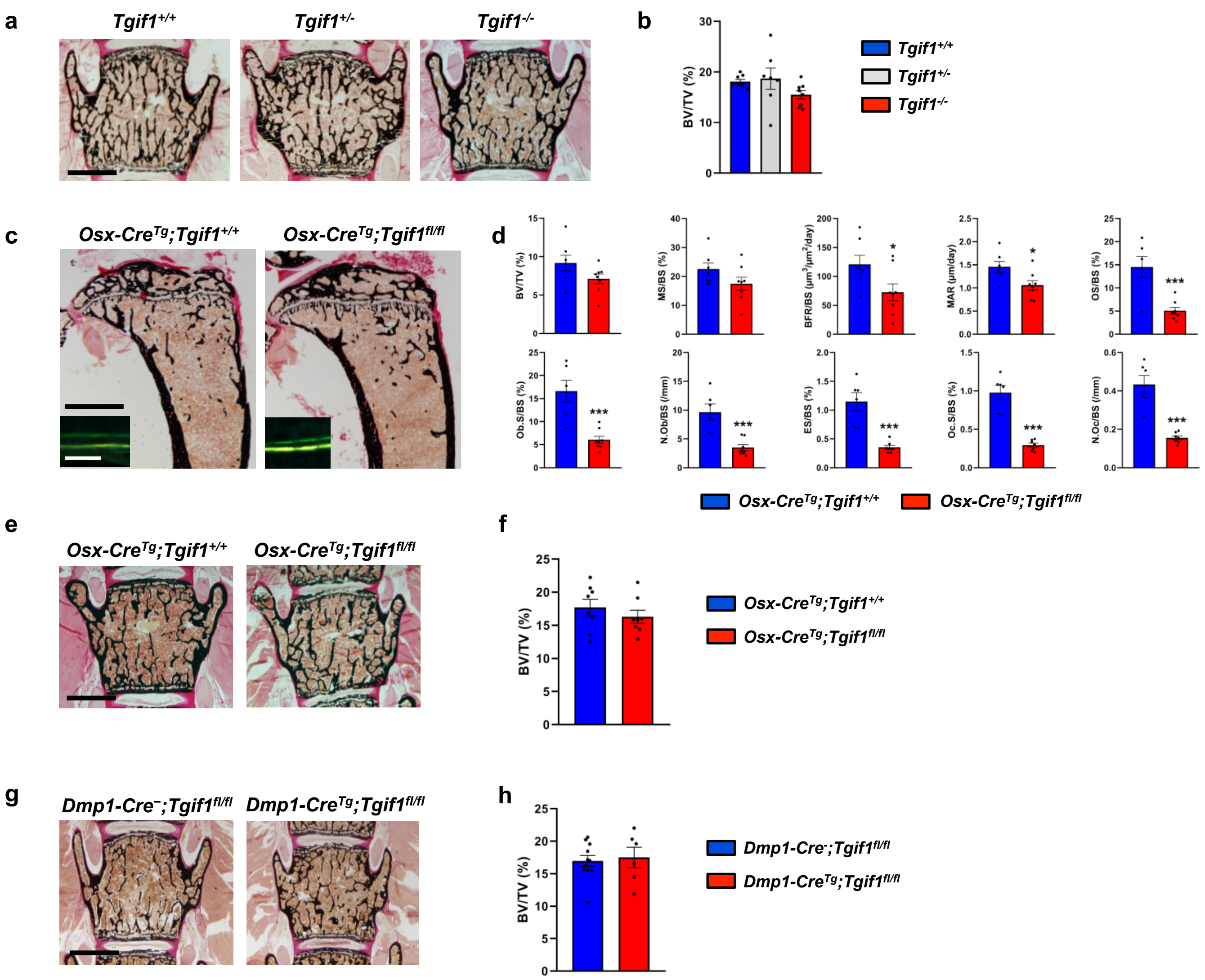

Supplementary Fig. 2 TG-interacting factor 1 (Tgif1)-deficiency in osteoblasts attenuates bone remodeling. **a** Representative images of the fourth lumbar vertebrae of 8-week-old male mice with the genotypes *Tgif1*$^{+/+}$, *Tgif1*$^{+/-}$ and *Tgif1*$^{-/-}$ after von Kossa staining. **b** Histomorphometric quantification of the bone mass (BV/TV, bone volume/tissue volume) of the fourth lumbar vertebrae of 8-week-old male mice with the genotypes *Tgif1*$^{+/+}$, *Tgif1*$^{+/-}$ and *Tgif1*$^{-/-}$ (N = 9, 7 and 8). **c** Representative images of the proximal tibiae of 8-week-old male mice of the genotypes *Osx-Cre*^{Tg}*;Tgif1*$^{+/+}$ and *Osx-Cre*^{Tg}*;Tgif1*$^{fl/fl}$, i.e. deletion of the *Tgif1* gene in osteoblasts of early differentiation stages, after von Kossa staining and fluorescence double labeling to visualize bone formation (insets). **d** Histomorphometric analysis of the proximal tibiae of 8-week-old male mice with the genotypes *Osx-Cre*^{Tg}*;Tgif1*$^{+/+}$ and *Osx-Cre*^{Tg}*;Tgif1*$^{fl/fl}$ (N = 7, 8). For abbreviations see the legend to Fig. 1. *p<0.05, ***p<0.001 vs. *Osx-Cre*^{Tg}*;Tgif1*$^{+/+}$. **e** Representative images of the fourth lumbar vertebrae of 8-week-old male mice with the genotypes *Osx-Cre*^{Tg}*;Tgif1*$^{+/+}$ and *Osx-Cre*^{Tg}*;Tgif1*$^{fl/fl}$ after von Kossa staining. **f** Histomorphometric quantification of the BV/TV of the fourth lumbar vertebrae of 8-week-old male mice with the genotypes *Osx-Cre*^{Tg}*;Tgif1*$^{+/+}$ and *Osx-Cre*^{Tg}*;Tgif1*$^{fl/fl}$ (N = 8, 8). **g** Representative images of the fourth lumbar vertebrae of 8-week-old male mice with the genotypes *Dmp1-Cre*$^{-}$*;Tgif1*$^{fl/fl}$ and *Dmp1-Cre*^{Tg}*;Tgif1*$^{fl/fl}$, i.e. deletion of the *Tgif1* gene in mature osteoblasts and osteocytes, after von Kossa staining. **h** Histomorphometric quantification of the BV/TV of the fourth lumbar vertebrae of 8-week-old male mice with the genotypes *Dmp1-Cre*$^{-}$*;Tgif1*$^{fl/fl}$ and *Dmp1-Cre*^{Tg}*;Tgif1*$^{fl/fl}$ (N = 11, 6). Scale bars indicate 1 mm **a**, **e**, **g**, and 1 mm (black) and 50 µm (white) **c**. Error bars represent the s.e.m. Two-tailed Student´s t-test was used to compare two groups **d**, **f**, **h**, and analysis of variance (ANOVA) followed by Newman-Keuls post-hoc analysis was used to compare three groups **b**

Supplementary Figure 3

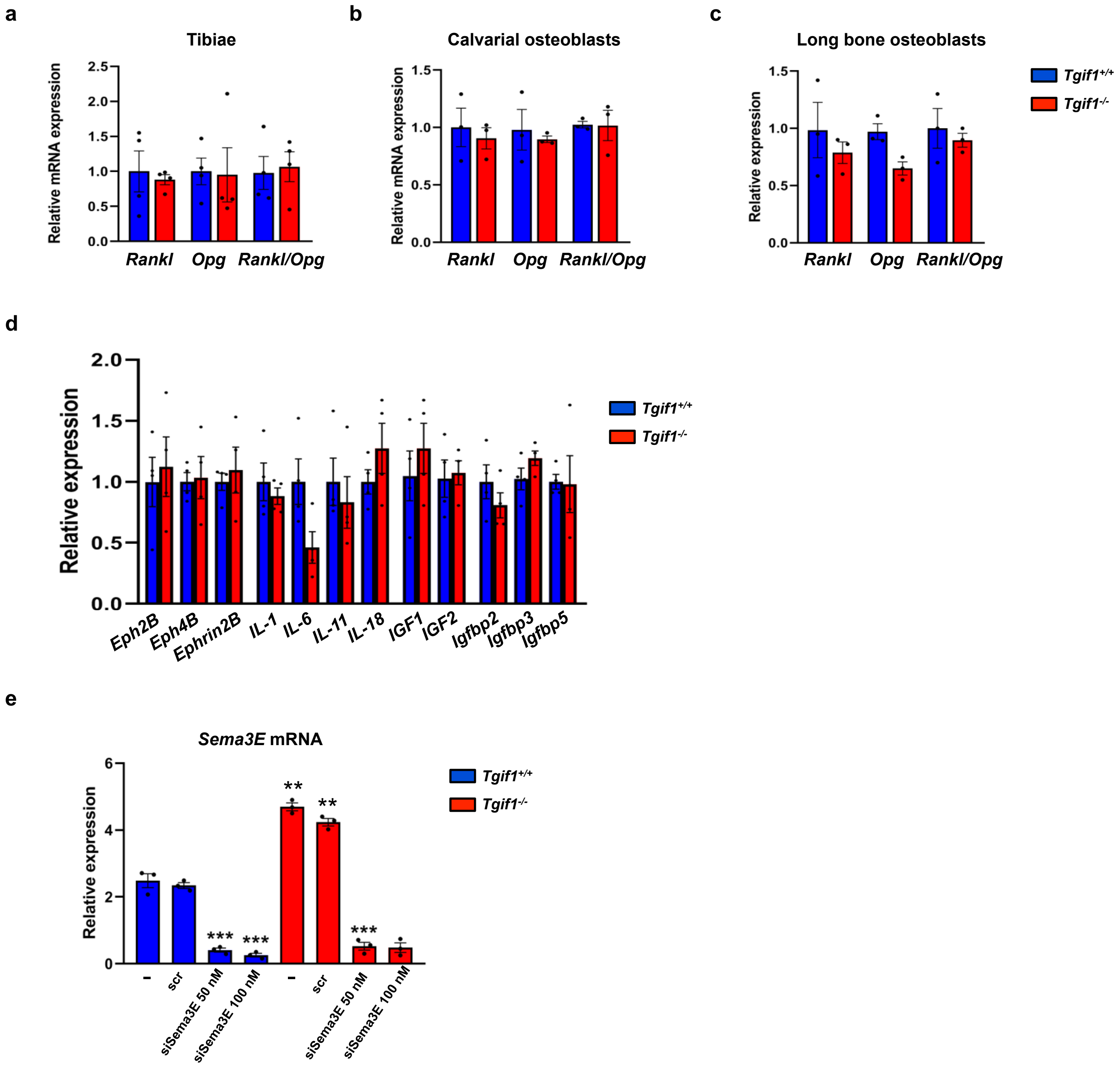

Supplementary Fig. 3 *Semaphorin 3E* (*Sema3E)* expression is increased in *TG-interacting factor 1* (*Tgif1)*-deficient osteoblasts and suppressed by siRNA. Relative *Rankl* and *Opg* mRNA expression and the calculated *Rankl*/*Opg* ratio in **a** tibiae**, b** calvarial osteoblasts and **c** long bone osteoblasts obtained from $Tgif1^{+/+}$ and $Tgif1^{-/-}$ mice (N = 4, 4). **d** Relative mRNA expression of indicated genes in long bone osteoblasts isolated from $Tgif1^{+/+}$ and $Tgif1^{-/-}$ mice (N = 4, 4). **e** Relative *Sema3E* mRNA expression in long bone osteoblasts isolated from $Tgif1^{+/+}$ and $Tgif1^{-/-}$ mice 24 hours after transfection with Sema3E siRNA (siSema3E) or scrambled (scr) control siRNA at a final concentration of 50 nM or 100 nM (N = 3). **p<0.01, ***p<0.001 vs. $Tgif1^{+/+}$ scr. Error bars represent the s.e.m. Two-tailed Student´s t-test was used to compare two groups **a**-**d**, and analysis of variance (ANOVA) followed by Newman-Keuls post-hoc analysis was used to compare more than two groups **e**

Supplementary Figure 4

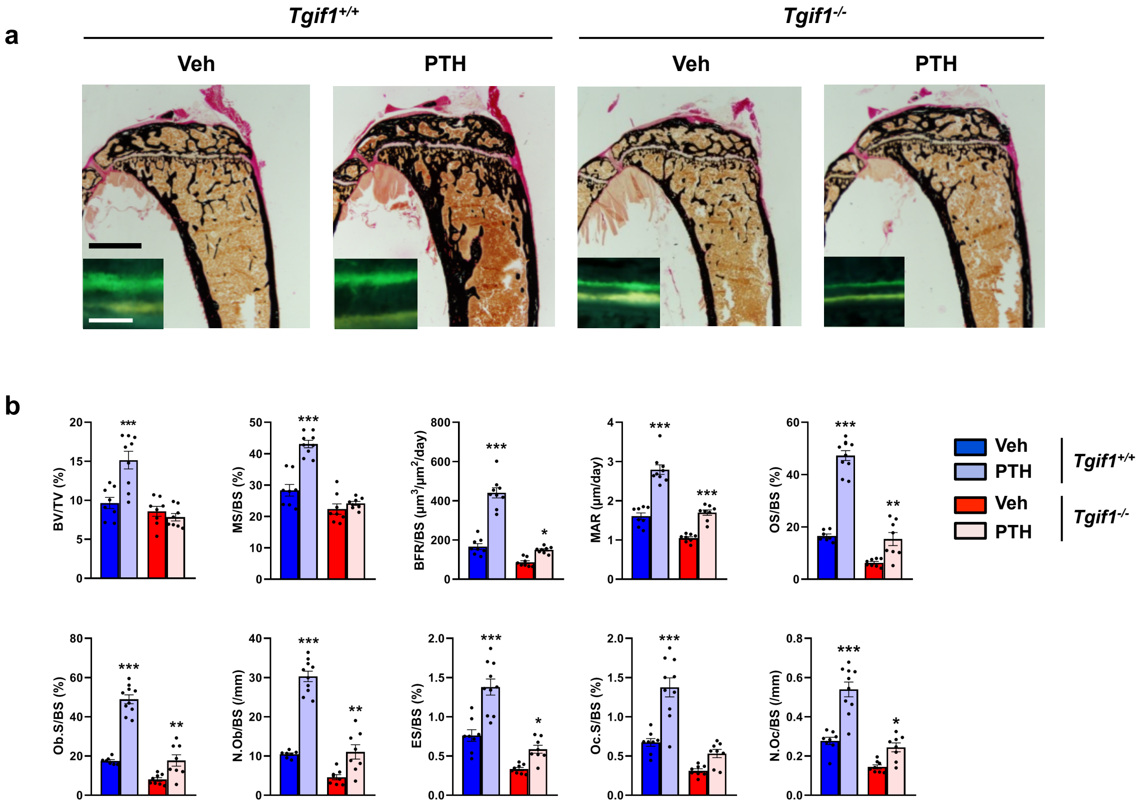

Supplementary Fig. 4 Deletion of TG-interacting factor 1 (Tgif1*)* blunts the bone anabolic effect of Parathyroid hormone (PTH) treatment. **a** Representative images of the proximal tibiae of 12-week-old *Tgif1*$^{+/+}$ and *Tgif1*$^{-/-}$ male mice after von Kossa staining and fluorescence double labeling to visualize bone formation (insets). *Tgif1*$^{+/+}$ and *Tgif1*$^{-/-}$ mice were treated with PTH or vehicle (Veh) for 4 weeks. Scale bars indicate 1 mm (black) and 50 µm (white). **b** Histomorphometric analysis of the trabecular bone in proximal tibiae of 12-week-old male *Tgif1*$^{+/+}$ and *Tgif1*$^{-/-}$ mice after treatment with PTH or Veh (*Tgif1*$^{+/+}$+Veh: N = 8, *Tgif1*$^{+/+}$+PTH: N = 9, *Tgif1*$^{-/-}$+Veh: N = 8, *Tgif1*$^{-/-}$+PTH: N = 8). For abbreviations see the legend to Fig. 1. *p<0.05, **p<0.01, ***p<0.001 vs. Veh of the same genotype. Error bars represent the s.e.m. Statistical analysis was performed using analysis of variance (ANOVA) followed by Newman-Keuls post-hoc test

Supplementary Figure 5

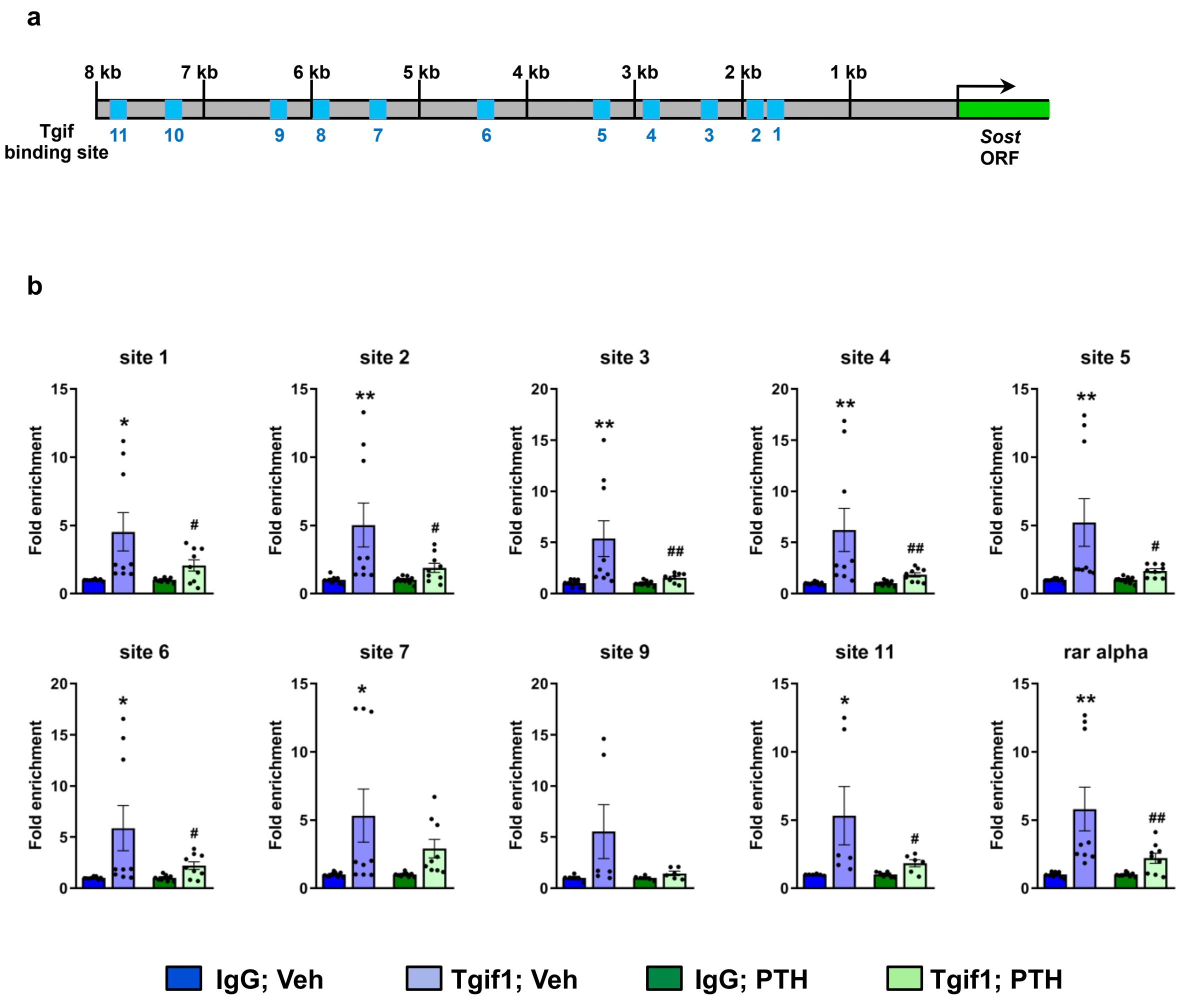

Supplementary Fig. 5 TG-interacting factor 1 (Tgif1) interacts with the *Sost* promoter and dissociates upon parathyroid hormone (PTH) stimulation. **a** Schematic of the 8 kb region of the *Sost* promoter upstream of the open reading frame (ORF) with 11 putative Tgif (Tgif1 and Tgif2) binding sites (marked in blue). (**b**) Chromatin-Immunoprecipitation (ChIP) was used to determine the fold enrichment of Tgif1 at the indicated sites of the *Sost* promoter in Ocy454 cells upon stimulation with PTH or vehicle (Veh) (N = 3). ChIP using an antibody against immunoglobulin G (IgG) was used as negative control and ChIP of Tgif1 with the *Rar alpha* promoter served as positive control. *p<0.05, **p<0.01 vs. IgG; Veh, $^{\#}$p<0.05, $^{\#\#}$p<0.05 vs. Tgif1; Veh. Error bars represent the s.e.m. Statistical analysis was performed using analysis of variance (ANOVA) followed by Newman-Keuls post-hoc test

Supplementary Figure 6

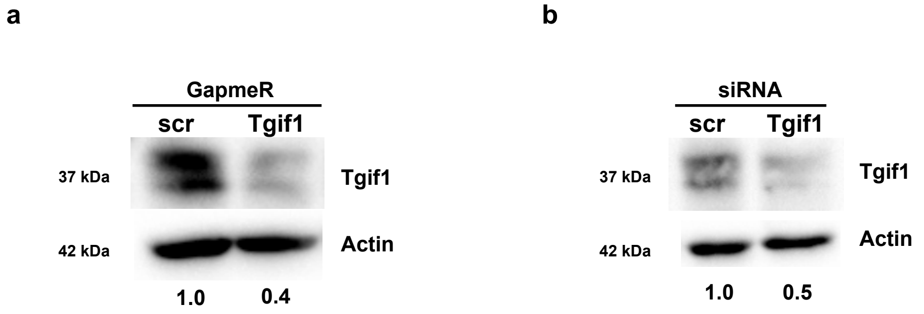

Supplementary Fig. 6 Silencing of TG-interacting factor 1 (Tgif1). **a** Immunoblot of Tgif1 protein expression in UMR-106 cells 48 hours after transfection with scrambled (scr) control GapmeR or GapmeR targeting Tgif1 or **b** with scr siRNA or siRNA targeting Tgif1. Immunoblot for Actin was used as a loading control. Normalized fold expression and molecular weight in kilo Dalton (kDa) are indicated (representative images of 3 experiments)

Supplementary Table 1. Histomorphometric analysis of the proximal tibiae and vertebrae of *Tgif1*$^{+/+}$, *Tgif1*$^{+/-}$ and *Tgif1*$^{-/-}$ male mice

	Parameters	Male		
		Tgif1$^{+/+}$	*Tgif1*$^{+/-}$	*Tgif1*$^{-/-}$
Proximal tibia	BV/TV (%)	8.666 ± 0.683 (n=9)	9.228 ± 0.780 (n=6)	7.527 ± 0.726 (n=7)
	Tb.Th (μm)	28.49 ± 2.63 (n=9)	32.36 ± 2.32 (n=6)	29.96 ± 1.02 (n=7)
	Tb.Sp (μm)	277.6 ± 45.2 (n=9)	328.2 ± 33.1 (n=6)	360.0 ± 49.1 (n=7)
	Tb.N (1/mm)	3.184 ± 0.461 (n=8)	2.892 ± 0.254 (n=6)	2.584 ± 0.238 (n=7)
	MS/BS (%)	16.62 ± 3.41 (n=9)	18.02 ± 2.54 (n=5)	9.659 ± 3.376 (n=7)
	MAR (μm/day)	1.693 ± 0.143 (n=9)	1.687 ± 0.186 (n=5)	0.957 ± 0.143**## (n=7)
	BFR/BS (μm^3/μm^2/year)	105.4 ± 21.1 (n=9)	109.4 ± 14.9 (n=5)	34.66 ± 12.2*# (n=7)
	BFR/BV (%/year)	589.4 ± 123.5 (n=9)	588.5 ± 88.7 (n=5)	183.0 ± 60.6* (n=7)
	OV/BV (%)	1.972 ± 0.207 (n=10)	1.910 ± 0.244 (n=6)	0.984 ± 0.202**# (n=8)
	OS/BS (%)	15.15 ± 0.80 (n=10)	12.34 ± 1.73 (n=6)	6.15 ± 0.74***### (n=8)
	Ob.S/BS (%)	17.85 ± 0.85 (n=10)	13.94 ± 1.98 (n=6)	7.91 ± 1.36***## (n=8)
	N.Ob/BS (1/mm)	10.67 ± 0.60 (n=10)	8.03 ± 1.12* (n=6)	4.71 ± 0.71***# (n=8)
	ES/BS (%)	0.9010 ± 0.0896 (n=10)	0.6667 ± 0.0919 (n=6)	0.3525 ± 0.0.0568***# (n=8)
	Oc.S/BS (%)	0.7910 ± 0.0690 (n=10)	0.5450 ± 0.0681* (n=6)	0.3188 ± 0.0396***# (n=8)
	N.Oc/BS (1/mm)	0.3550 ± 0.0300 (n=10)	0.2567 ± 0.0232* (n=6)	0.1688 ± 0.0245*** (n=8)
Vertebral body	BV/TV (%)	18.93 ± 0.90 (n=9)	18.68 ± 2.09 (n=7)	15.50 ± 0.81 (n=8)
	Tb.Th (μm)	39.29 ± 0.90 (n=9)	38.60 ± 2.09 (n=7)	35.52 ± 1.10 (n=8)
	Tb.Sp (μm)	169.1 ± 3.9 (n=9)	177.7 ± 18.4 (n=7)	195.9 ± 7.9 (n=8)
	Tb.N (1/mm)	4.806 ± 0.070 (n=8)	4.757 ± 0.295 (n=11)	4.353 ± 0.140 (n=8)

Histomorphometry of the proximal tibiae and the L4 vertebral bodies of 8-week old mice. Mean values ± SEM. *p<0.05, **p<0.01, ***p<0.001 vs. *Tgif1*$^{+/+}$, #p<0.05, ##p<0.01, ###p<0.001 vs. *Tgif1*$^{+/-}$.

Supplementary Table 2. Histomorphometric analysis of the proximal tibiae and vertebrae of *Tgif1*$^{+/+}$, *Tgif1*$^{+/-}$ and *Tgif1*$^{-/-}$ female mice

	Parameters	Female		
		Tgif1$^{+/+}$	*Tgif1*$^{+/-}$	*Tgif1*$^{-/-}$
Proximal tibia	BV/TV (%)	5.321 ± 0.764 (n=7)	5.540 ± 0.546 (n=8)	5.224 ± 0.466 (n=7)
	Tb.Th (μm)	31.52 ± 2.20 (n=7)	27.57 ± 1.52 (n=8)	28.09 ± 2.68 (n=7)
	Tb.Sp (μm)	592.8 ± 63.1 (n=7)	495.5 ± 45.1 (n=8)	547.0 ± 82.9 (n=7)
	Tb.N (1/mm)	1.626 ± 0.170 (n=7)	1.993 ± 0.139 (n=8)	2.059 ± 0.408 (n=8)
	MS/BS (%)	23.16 ± 3.81 (n=7)	14.97 ± 0.88 (n=7)	15.31 ± 2.82 (n=7)
	MAR (μm/day)	1.942 ± 0.070 (n=7)	1.607 ± 0.120 (n=7)	1.019 ± 0.178***$^{\#}$ (n=7)
	BFR/BS (μm^3/μm^2/year)	159.6 ± 23.4 (n=7)	87.78 ± 8.02* (n=7)	59.57 ± 12.85*** (n=7)
	BFR/BV (%/year)	837.1 ± 109.3 (n=7)	507.8 ± 52.6* (n=7)	340.7 ± 69.9** (n=7)
	OV/BV (%)	3.169 ± 0.490 (n=7)	4.223 ± 0.797 (n=8)	1.887 ± 0.566$^{\#}$ (n=7)
	OS/BS (%)	21.13 ± 1.37 (n=7)	20.90 ± 2.65 (n=8)	10.38 ± 2.21**$^{\#\#}$ (n=7)
	Ob.S/BS (%)	23.16 ± 1.23 (n=7)	26.04 ± 2.18 (n=8)	10.85 ± 1.24***$^{\#\#\#}$ (n=7)
	N.Ob/BS (1/mm)	13.34 ± 0.80 (n=7)	15.80 ± 1.29* (n=8)	6.51 ± 1.48**$^{\#\#\#}$ (n=7)
	ES/BS (%)	1.183 ± 0.117 (n=7)	0.9363 ± 0.139 (n=8)	0.4400 ± 0.0628***$^{\#}$ (n=7)
	Oc.S/BS (%)	1.104 ± 0.144 (n=7)	0.7688 ± 0.1139 (n=8)	0.3686 ± 0.0639*** (n=7)
	N.Oc/BS (1/mm)	0.5157 ± 0.0395 (n=7)	0.3563 ± 0.0479 (n=8)	0.2543 ± 0.0542** (n=7)
Vertebral body	BV/TV (%)	14.37 ± 2.96 (n=3)	11.68 ± 1.15 (n=8)	11.42 ± 3.43 (n=4)
	Tb.Th (μm)	38.39 ± 2.49 (n=3)	33.57 ± 1.65 (n=8)	34.89 ± 4.07 (n=4)
	Tb.Sp (μm)	244.6 ± 41.5 (n=3)	264.5 ± 18.2 (n=8)	309.9 ± 49.6 (n=4)
	Tb.N (1/mm)	3.676 ± 0.522 (n=3)	3.425 ± 0.179 (n=8)	3.111 ± 0.528 (n=4)

Histomorphometry of the proximal tibiae and the L4 vertebral bodies of 8-week old mice. Mean values ± SEM. *p<0.05, **p<0.01, ***p<0.001 vs. *Tgif1*$^{+/+}$, $^{\#}$p<0.05, $^{\#\#}$p<0.01, $^{\#\#\#}$p<0.001 vs. *Tgif1*$^{+/-}$.

Supplementary Table 3. Histomorphometric analysis of the proximal tibiae and vertebrae of *Osx-Cre*^{Tg};*Tgif1*$^{+/+}$ and *Osx-Cre*^{Tg};*Tgif1*$^{fl/fl}$ male mice

		Male	
	Parameters	*Osx-Cre*^{Tg};*Tgif1*$^{+/+}$	*Osx-Cre*^{Tg};*Tgif1*$^{fl/fl}$
Proximal tibia	BV/TV (%)	9.178 ± 1.03 (n=7)	7.109 ± 0.63 (n=8)
	Tb.Th (μm)	33.31 ± 0.90 (n=7)	31.40 ± 1.03 (n=8)
	Tb.Sp (μm)	361.5 ± 54.0 (n=7)	438.7 ± 49.4 (n=8)
	Tb.N (1/mm)	2.770 ± 0.306 (n=7)	2.256 ± 0.181 (n=8)
	MS/BS (%)	22.51 ± 2.09 (n=7)	17.49 ± 2.23 (n=8)
	MAR (μm/day)	1.457 ± 0.113 (n=7)	1.059 ± 0.100* (n=8)
	BFR/BS (μm^3/μm^2/year)	120.9 ± 15.6 (n=7)	72.44 ± 14.6* (n=8)
	BFR/BV (%/year)	718.6 ± 79.6 (n=7)	467.4 ± 97.4 (n=8)
	OV/BV (%)	1.892 ± 0.384 (n=6)	0.591 ± 0.010** (n=8)
	OS/BS (%)	14.52 ± 2.28 (n=6)	5.063 ± 0.713*** (n=8)
	Ob.S/BS (%)	16.62 ± 2.34 (n=6)	6.044 ± 0.768*** (n=8)
	N.Ob/BS (1/mm)	9.648 ± 1.407 (n=6)	3.484 ± 0.509*** (n=8)
	ES/BS (%)	1.149 ± 0.155 (n=6)	0.432 ± 0.083*** (n=8)
	Oc.S/BS (%)	0.9764 ± 0.0936 (n=6)	0.3671 ± 0.0780*** (n=8)
	N.Oc/BS (1/mm)	0.4335 ± 0.0472 (n=6)	0.1864 ± 0.0325*** (n=8)
Vertebral body	BV/TV (%)	17.69 ± 1.23 (n=8)	16.30 ± 0.97 (n=8)
	Tb.Th (μm)	36.00 ± 1.49 (n=8)	36.17 ± 1.04 (n=8)
	Tb.Sp (μm)	172.3 ± 11.8 (n=8)	189.1 ± 10.1 (n=8)
	Tb.N (1/mm)	4.908 ± 0.277 (n=8)	4.493 ± 0.182 (n=11)

Histomorphometry of the proximal tibiae and the L4 vertebral bodies of 8-week old mice. Mean values ± SEM. *p<0.05, **p<0.01, ***p<0.001 vs. *Osx-Cre*^{Tg};*Tgif1*$^{+/+}$.

Supplementary Table 4. Histomorphometric analysis of the proximal tibiae and vertebrae of *Dmp1-Cre*$^{-}$;*Tgif1*$^{fl/fl}$ and *Dmp1-Cre*^{Tg};*Tgif1*$^{fl/fl}$ male mice

		Male	
	Parameters	*Dmp1-Cre*$^{-}$;*Tgif1*$^{fl/fl}$	*Dmp1-Cre*^{Tg};*Tgif1*$^{fl/fl}$
Proximal tibia	BV/TV (%)	5.761 ± 0.825 (n=9)	6.176 ± 0.680 (n=8)
	Tb.Th (µm)	25.60 ± 1.31 (n=9)	26.62 ± 2.02 (n=8)
	Tb.Sp (µm)	503.2 ± 79.0 (n=9)	443.5 ± 64.6 (n=8)
	Tb.N (1/mm)	2.283 ± 0.340 (n=9)	2.353 ± 0.245 (n=8)
	MS/BS (%)	27.77 ± 0.87 (n=9)	20.71 ± 1.26*** (n=8)
	MAR (µm/day)	1.924 ± 0.101 (n=9)	1.092 ± 0.037*** (n=8)
	BFR/BS (µm^{3}/µm^{2}/year)	194.8 ± 11,2 (n=9)	82.25 ± 5.02*** (n=8)
	BFR/BV (%/year)	1123.0 ± 83.3 (n=9)	487.5 ± 35.2*** (n=8)
	OV/BV (%)	2.762 ± 0.205 (n=8)	0.870 ± 0.335*** (n=6)
	OS/BS (%)	16.87 ± 1.34 (n=8)	6.474 ± 2.267** (n=6)
	Ob.S/BS (%)	17.63 ± 1.26 (n=8)	7.769 ± 2.853** (n=6)
	N.Ob/BS (1/mm)	11.55 ± 0.77 (n=8)	5.206 ± 1.780** (n=6)
	ES/BS (%)	0.9783 ± 0.0674 (n=8)	0.4811 ± 0.0685*** (n=6)
	Oc.S/BS (%)	0.8966 ± 0.0554 (n=8)	0.4913 ± 0.0852** (n=6)
	N.Oc/BS (1/mm)	0.3684 ± 0.0264 (n=8)	0.2160 ± 0.0382** (n=6)
Vertebral body	BV/TV (%)	17.54 ± 1.12 (n=18)	18.13 ± 2.01 (n=9)
	Tb.Th (µm)	30.53 ± 1.52 (n=18)	28.56 ± 2.53 (n=9)
	Tb.Sp (µm)	149.9 ± 8.7 (n=18)	189.1 ± 10.1 (n=9)
	Tb.N (1/mm)	5.728 ± 0.239 (n=18)	6.250 ± 0.361 (n=9)

Histomorphometry of the proximal tibiae and the L4 vertebral bodies of 8-week old mice. Mean values ± SEM. **p<0.01, ***p<0.001 vs. *Dmp1-Cre*$^{-}$;*Tgif1*$^{fl/fl}$.

Supplementary Table 5. Histomorphometric analysis of the proximal tibiae of *Tgif1*$^{+/+}$ and *Tgif1*$^{-/-}$ male mice after Scl-Ab treatment

		Male			
	Parameters	*Tgif1*$^{+/+}$, vehicle	*Tgif1*$^{+/+}$, Scl-Ab	*Tgif1*$^{-/-}$, vehicle	*Tgif1*$^{-/-}$, Scl-Ab
Proximal tibia	BV/TV (%)	7.726 ± 0.894 (n=11)	17.19 ± 1.35*** (n=9)	7.410 ± 0.678 (n=6)	15.95 ± 1.46*** (n=8)
	Tb.Th (μm)	28.23 ± 1.98 (n=11)	51.29 ± 3.32*** (n=9)	28.02 ± 1.62 (n=6)	46.76 ± 1.50*** (n=8)
	Tb.Sp (μm)	355.1 ± 21.3 (n=11)	258.0 ± 28.5 (n=9)	357.9 ± 21.7 (n=6)	270.2 ± 39.4 (n=8)
	Tb.N (1/mm)	2.673 ± 0.126 (n=11)	3.411 ± 0.235 (n=9)	2.629 ± 0.140 (n=6)	3.436 ± 0.322 (n=8)
	MS/BS (%)	27.49 ± 2.36 (n=12)	46.76 ± 2.80*** (n=9)	22.88 ± 3.52 (n=6)	50.87 ± 3.88*** (n=8)
	MAR (μm/day)	1.697 ± 0.063 (n=12)	2.124 ± 0.128** (n=9)	0.9904 ± 0.0668 (n=6)	1.895 ± 0.115*** (n=8)
	BFR/BS ($\mu m^3/\mu m^2$/year)	169.5 ± 14.4 (n=12)	356.4 ± 19.8*** (n=9)	83.83 ± 15.4 (n=6)	347.6 ± 27.3*** (n=8)
	BFR/BV (%/year)	877.9 ± 92.8 (n=12)	1138 ± 70.5 (n=9)	443.5 ± 77.9 (n=6)	1123 ± 77.1*** (n=8)
	OV/BV (%)	1.546 ± 0.201 (n=8)	4.307 ± 0.500*** (n=8)	0.6648 ± 0.0931 (n=6)	2.685 ± 0.452*** (n=8)
	OS/BS (%)	9.834 ± 1.002 (n=8)	30.77 ± 3.57*** (n=8)	5.134 ± 0.718 (n=6)	22.51 ± 1.81*** (n=8)
	Ob.S/BS (%)	10.25 ± 1.06 (n=8)	31.29 ± 3.32*** (n=8)	5.599 ± 1.009 (n=6)	24.36 ± 1.73*** (n=8)
	N.Ob/BS (1/mm)	5.904 ± 0.560 (n=8)	17.18 ± 1.67*** (n=8)	3.650 ± 0.720 (n=6)	13.00 ± 1.31*** (n=8)
	ES/BS (%)	0.7151 ± 0.0708 (n=8)	0.7905 ± 0.0565 (n=8)	0.3818 ± 0.0364 (n=6)	0.4523 ± 0.0426 (n=8)
	Oc.S/BS (%)	0.6741 ± 0.0733 (n=8)	0.7948 ± 0.0716 (n=8)	0.3203 ± 0.0300 (n=6)	0.4719 ± 0.0506 (n=8)
	N.Oc/BS (1/mm)	0.2864 ± 0.0276 (n=8)	0.3190 ± 0.0321 (n=8)	0.1418 ± 0.0116 (n=6)	0.1890 ± 0.0216 (n=8)

Histomorphometry of the proximal tibiae of 12-week old mice. Mean values ± SEM. **$p<0.01$, ***$p<0.001$ vs. vehicle control of the same genotype.

Supplementary Table 6. Histomorphometric analysis of the proximal tibiae of *Tgif1*$^{+/+}$ and *Tgif1*$^{-/-}$ male mice with *Lrp5* $^{+/G171V}$ high bone mass mutation

		Male			
	Parameters	*Lrp5* $^{+/+}$;*Tgif1*$^{+/+}$	*Lrp5* $^{+/G171V}$;*Tgif1*$^{+/+}$	*Lrp5* $^{+/+}$;*Tgif1*$^{-/-}$	*Lrp5* $^{+/G171V}$;*Tgif1*$^{-/-}$
Proximal tibia	BV/TV (%)	6.702 ± 0.867 (n=8)	16.40 ± 1.64*** (n=8)	6.195 ± 0.700 (n=8)	10.62 ± 1.13* (n=6)
	Tb.Th (μm)	29.97 ± 2.32 (n=8)	53.69 ± 3.54*** (n=8)	29.20 ± 1.66 (n=8)	40.30 ± 2.92** (n=6)
	Tb.Sp (μm)	408.5 ± 29.8 (n=8)	283.0 ± 20.4* (n=8)	469.8 ± 42.8 (n=8)	350.3 ± 27.7 (n=6)
	Tb.N (1/mm)	2.358 ± 0.147 (n=8)	3.038 ± 0.170* (n=8)	2.098 ± 0.165 (n=8)	2.622 ± 0.182 (n=6)
	MS/BS (%)	33.62 ± 1.62 (n=8)	44.63 ± 2.83** (n=8)	23.46 ± 1.83 (n=8)	39.74 ± 1.10*** (n=6)
	MAR (μm/day)	1.818 ± 0.149 (n=8)	2.639 ± 0.197** (n=8)	1.462 ± 0.149 (n=8)	2.512 ± 0.138*** (n=6)
	BFR/BS ($\mu m^3/\mu m^2$/year)	227.8 ± 29.2 (n=8)	441.2 ± 61.0** (n=8)	129.8 ± 23.4 (n=8)	365.1 ± 25.3** (n=6)
	BFR/BV (%/year)	1126 ± 104 (n=8)	1347 ± 140 (n=8)	776.6 ± 122.7 (n=8)	1709 ± 172*** (n=6)
	OV/BV (%)	2.278 ± 0.408 (n=8)	3.252 ± 0.405 (n=8)	1.109 ± 0.257 (n=8)	3.514 ± 0.422** (n=6)
	OS/BS (%)	13.55 ± 1.71 (n=8)	25.40 ± 2.04*** (n=8)	7.116 ± 1.175 (n=8)	21.43 ± 1.21*** (n=6)
	Ob.S/BS (%)	14.33 ± 1.71 (n=8)	25.99 ± 2.26*** (n=8)	8.672 ± 1.545 (n=8)	22.19 ± 1.28*** (n=6)
	N.Ob/BS (1/mm)	9.010 ± 0.950 (n=8)	16.14 ± 1.52*** (n=8)	5.512 ± 0.978 (n=8)	13.01 ± 0.92*** (n=6)
	ES/BS (%)	1.029 ± 0.071 (n=8)	1.153 ± 0.120 (n=8)	0.4937 ± 0.0703 (n=8)	0.6011 ± 0.0876 (n=6)
	Oc.S/BS (%)	0.9342 ± 0.0492 (n=8)	1.079 ± 0.108 (n=8)	0.4385 ± 0.0614 (n=8)	0.5242 ± 0.0862 (n=6)
	N.Oc/BS (1/mm)	0.3740 ± 0.0210 (n=8)	0.3933 ± 0.0506 (n=8)	0.2007 ± 0.0237 (n=8)	0.2125 ± 0.0363 (n=6)

Histomorphometry of the proximal tibiae of 8-week old mice. Mean values ± SEM. *p<0.05, **p<0.01, ***p<0.001 vs. group with the same Tgif1 genotype.

Supplementary Table 7. µCT analysis of the distal and midshaft femura of *Tgif1*$^{+/+}$ and *Tgif1*$^{-/-}$ male mice with *Lrp5* $^{+/A214V}$ high bone mass mutation

		Male			
	Parameters	*Lrp5*$^{+/+}$;*Tgif1*$^{+/+}$	*Lrp5*$^{+/A214V}$;*Tgif1*$^{+/+}$	*Lrp5*$^{+/+}$;*Tgif1*$^{-/-}$	*Lrp5*$^{+/A214V}$;*Tgif1*$^{-/-}$
Distal femur	BV/TV (%)	28.32 ± 6.28 (n=8)	54.13 ± 2.89** (n=9)	23.81 ± 6.05 (n=7)	43.74 ± 3.07** (n=7)
	Tb.Th (µm)	52.00 ± 7.11 (n=8)	92.71 ± 6.94*** (n=9)	49.76 ± 9.07 (n=7)	74.84 ± 5.78 (n=7)
	Tb.Sp (µm)	179.6 ± 42.4 (n=8)	77.34 ± 4.06 (n=9)	208.5 ± 53.5 (n=7)	95.94 ± 5.15 (n=7)
	Tb.N (1/mm)	4.931 ± 0.581 (n=8)	5.244 ± 0.605 (n=9)	4.446 ± 0.533 (n=7)	5.862 ± 0.083 (n=7)
	SMI	1.150 ± 0.658 (n=8)	-1.950 ± 0.620** (n=9)	1.573 ± 0.633 (n=7)	-0.263 ± 0.439 (n=7)
Midshaft femur	Ct.Th (µm)	188.1 ± 6.4 (n=8)	242.2 ± 4.0*** (n=9)	179.0 ± 11.0 (n=7)	231.4 ± 12.6*** (n=7)
	Ct.Dens (mg HA/cm^3)	915.4 ± 62.3 (n=8)	1071 ± 12 (n=9)	927.1 ± 62.4 (n=7)	975.7 ± 19.4 (n=7)
	Ps.Dm (mm)	1.858 ± 0.025 (n=8)	1.943 ± 0.029 (n=9)	1.798 ± 0.038 (n=7)	1.906 ± 0.054 (n=7)
	Ps.Pm (mm)	5.835 ± 0.079 (n=8)	6.101 ± 0.091 (n=9)	5.645 ± 0.120 (n=7)	5.986 ± 0.170 (n=7)
	Ec.Dm (mm)	1.565 ± 0.024 (n=8)	1.568 ± 0.024 (n=9)	1.526 ± 0.027 (n=7)	1.551 ± 0.040 (n=7)
	Ec.Pm (mm)	4.913 ± 0.076 (n=8)	4.923 ± 0.074 (n=9)	4.792 ± 0.085 (n=7)	4.872 ± 0.126 (n=7)

µCT analysis of the distal femura of 8-week old mice. BV/TV, bone volume/total volume; Tb.Th, trabecular thickness; Tb.Sp, trabecular separation; Tb.N, trabecular number; SMI, structure model index; Ct.Th, cortical thickness; Ct.Dens, cortical density; Ps.Dm, periosteal diameter; Ps.Pm, periosteal perimeter; Ec.Dm, endocortical diameter; Ec.Pm, endocortical perimeter. Mean values ± SEM. **p<0.01, ***p<0.001 vs. group with the same Tgif1 genotype.

Supplementary Table 8. Histomorphometric analysis of the proximal tibiae of *Tgif1*$^{+/+}$ and *Tgif1*$^{-/-}$ male mice after anabolic PTH treatment

		Male			
	Parameters	*Tgif1*$^{+/+}$, vehicle	*Tgif1*$^{+/+}$, PTH	*Tgif1*$^{-/-}$, vehicle	*Tgif1*$^{-/-}$, PTH
Proximal tibia	BV/TV (%)	9.649 ± 0.718 (n=8)	15.14 ± 1.13*** (n=9)	8.589 ± 0.632 (n=8)	7.632 ± 0.476 (n=8)
	Tb.Th (μm)	38.69 ± 2.28 (n=8)	53.13 ± 2.85*** (n=9)	35.67 ± 1.72 (n=8)	38.33 ± 1.45 (n=8)
	Tb.Sp (μm)	371.4 ± 25.6 (n=8)	307.0 ± 20.8* (n=9)	388.8 ± 22.4 (n=8)	456.6 ± 16.3* (n=8)
	Tb.N (1/mm)	2.507 ± 0.159 (n=8)	2.843 ± 0.149 (n=9)	2.399 ± 0.120 (n=8)	2.036 ± 0.071 (n=8)
	MS/BS (%)	28.35 ± 1.87 (n=8)	43.08 ± 1.19*** (n=9)	22.36 ± 1.63 (n=8)	24.16 ± 0.62 (n=8)
	MAR (μm/day)	1.606 ± 0.086 (n=8)	2.794 ± 0.123*** (n=9)	1.048 ± 0.037 (n=8)	1.699 ± 0.069*** (n=8)
	BFR/BS (μm^3/μm^2/year)	166.4 ± 14.9 (n=8)	440.8 ± 26.0*** (n=9)	86.12 ± 8.02 (n=8)	149.3 ± 5.8* (n=8)
	BFR/BV (%/year)	860.3 ± 59.4 (n=8)	1664 ± 60.1*** (n=9)	502.6 ± 68.4 (n=8)	782.5 ± 29.5** (n=8)
	OV/BV (%)	3.285 ± 0.558 (n=8)	6.998 ± 0.397*** (n=9)	0.8817 ± 0.0815 (n=8)	1.983 ± 0.374 (n=8)
	OS/BS (%)	18.53 ± 2.09 (n=8)	47.30 ± 1.90*** (n=9)	6.286 ± 0.515 (n=8)	15.36 ± 2.48** (n=8)
	Ob.S/BS (%)	19.29 ± 1.90 (n=8)	48.92 ± 2.27*** (n=9)	8.008 ± 0.935 (n=8)	17.70 ± 2.89** (n=8)
	N.Ob/BS (1/mm)	11.38 ± 0.99 (n=8)	30.29 ± 1.35*** (n=9)	4.592 ± 0.648 (n=8)	11.03 ± 1.82** (n=8)
	ES/BS (%)	0.7615 ± 0.0744 (n=8)	1.379 ± 0.103*** (n=9)	0.3329 ± 0.0210 (n=8)	0.5862 ± 0.0511* (n=8)
	Oc.S/BS (%)	0.6730 ± 0.0497 (n=8)	1.375 ± 0.122*** (n=9)	0.3130 ± 0.0239 (n=8)	0.5312 ± 0.0526 (n=8)
	N.Oc/BS (1/mm)	0.2768 ± 0.0189 (n=8)	0.5396 ± 0.0375*** (n=9)	0.1446 ± 0.0103 (n=8)	0.2439 ± 0.0226* (n=8)

Histomorphometry of the proximal tibiae of 12-week old mice. Mean values ± SEM. *p<0.05, **p<0.01, ***p<0.001 vs. vehicle control of the same genotype.

Supplementary Table 9. Histomorphometric analysis of the proximal tibiae of *Dmp1-Cre*$^{-}$;*Tgif1*$^{fl/fl}$ and *Dmp1-Cre*^{Tg};*Tgif1*$^{fl/fl}$ male mice after anabolic PTH treatment

		Male			
	Parameters	*Dmp1-Cre*$^{-}$;*Tgif1*$^{fl/fl}$, vehicle	*Dmp1-Cre*$^{-}$;*Tgif1*$^{fl/fl}$, PTH	*Dmp1-Cre*^{Tg};*Tgif1*$^{fl/fl}$, vehicle	*Dmp1-Cre*^{Tg};*Tgif1*$^{fl/fl}$, PTH
Proximal tibia	BV/TV (%)	10.78 ± 0.94 (n=8)	18.69 ± 2.28** (n=8)	10.30 ± 1.10 (n=8)	11.68 ± 0.73 (n=8)
	Tb.Th (μm)	39.37 ± 2.68 (n=8)	48.49 ± 4.38 (n=8)	37.88 ± 2.51 (n=8)	44.37 ± 2.75 (n=8)
	Tb.Sp (μm)	331.5 ± 15.3 (n=8)	222.1 ± 20.2** (n=8)	350.1 ± 31.4 (n=8)	340.4 ± 20.81 (n=8)
	Tb.N (1/mm)	2.726 ± 0.109 (n=8)	3.820 ± 0.257*** (n=8)	2.674 ± 0.185 (n=8)	2.658 ± 0.150 (n=8)
	MS/BS (%)	29.26 ± 2.41 (n=8)	38.86 ± 2.51** (n=8)	20.09 ± 1.45 (n=8)	24.39 ± 2.39 (n=8)
	MAR (μm/day)	1.512 ± 0.106 (n=8)	2.598 ± 0.130*** (n=8)	1.348 ± 0.071 (n=8)	1.511 ± 0.162 (n=8)
	BFR/BS ($\mu m^3/\mu m^2$/year)	166.1 ± 23.7 (n=8)	366.6 ± 25.4*** (n=8)	97.92 ± 7.55 (n=8)	140.6 ± 28.4 (n=8)
	BFR/BV (%/year)	829.5 ± 63.2 (n=8)	1603 ± 37.9*** (n=8)	538.2 ± 52.2 (n=8)	737.7 ± 103.0* (n=8)
	OV/BV (%)	2.485 ± 0.226 (n=8)	7.972 ± 1.043*** (n=8)	0.6901 ± 0.0897 (n=8)	2.300 ± 0.449* (n=8)
	OS/BS (%)	15.83 ± 1.20 (n=8)	44.41 ± 4.64*** (n=8)	5.728 ± 0.766 (n=8)	15.54 ± 2.20* (n=8)
	Ob.S/BS (%)	16.56 ± 1.07 (n=8)	45.61 ± 4.80*** (n=8)	6.770 ± 0.843 (n=8)	17.77 ± 2.53* (n=8)
	N.Ob/BS (1/mm)	10.49 ± 0.62 (n=8)	30.65 ± 3.45*** (n=8)	4.505 ± 0.583 (n=8)	10.49 ± 1.43 (n=8)
	ES/BS (%)	0.7816 ± 0.0959 (n=8)	1.477 ± 0.166*** (n=8)	0.3506 ± 0.0342 (n=8)	0.7795 ± 0.0643** (n=8)
	Oc.S/BS (%)	0.7378 ± 0.0800 (n=8)	1.396 ± 0.179*** (n=8)	0.3563 ± 0.0393 (n=8)	0.7402 ± 0.0678* (n=8)
	N.Oc/BS (1/mm)	0.3045 ± 0.0322 (n=8)	0.5532 ± 0.0901** (n=8)	0.1651 ± 0.0128 (n=8)	0.3371 ± 0.0302 (n=8)

Histomorphometry of the proximal tibiae of 12-week old mice. Mean values ± SEM. *p<0.05, **p<0.01, ***p<0.001 vs. vehicle control of the same genotype.

Supplementary Table 10. Primer sequences

Primer name	Forward primer	Reverse primer
Alp	CCTCAAAGGCTTCTTCTTGCTG	GGGGTGTATCCACCGAATGTG
Ocn	CACTCTGCTGACCCTGGCTGC	CAGGGTTAAGCTCACACTGCTCC
Sema3E	GGGGCAGATGTCCTTTTGA	AGTCCAGCAAACAGCTCATTC
BAT-GAL	TTGAAAATGGTCTGCTGCTG	TATTGGCTTCATCCACCACA
CyclinD1	CGTGGCCTCTAAGATGAAGGA	TTG TTC TCA TCC GCC TCT GG
Axin2	GCAGCAGATCCGGGAGGATGAA	GATTGACAGCCGGGGGTCTTGA
Tgif1	GCAGACACACCTGTCCACACTA	GGAATGAAATGGGCTCTCTTCT
Tgif2	CAGACCAACCTCTCGGTGCTG	GCACACAGACAAGGCGAGCATG
Dkk1	CGAACAAGTACCGACTCT	GTCAGAGGGCATGCATACC
Sost	GAGAACAACCAGACCATGAAC	GCT CGC GGC AGC TGT ACT
Rankl	AACTGCAACACATTGTGGGC	TTATGGGAACCCGATGGGATGC
Opg	AAGAGCAAACCTTCCAGCTGC	CACGCTGCTTTCACAGAGGTC
Eph2B	CAGACCAGCATCAAGGAAAAG	ATGTGTCCGCTGGTGTAGTG
Eph4B	CACCCAGCAGCTTGATCCTG	ACCAGGACCACACCCACAAC
Ephrin2B	GTGCCAGACAAGAGCCATGAA	GGTGCTAGAACCTGGATTTGG
IL-1	GACCTTCCAGGATGAGGACA	AGGCCACAGGTATTTTGTCG
IL-6	GAGCCCACCAAGAACGATAG	GGTTGTCACCAGCATCAGTC
IL-11	CGGCAACTAGCTGCACAGATG	CTCCAGAGTCTTTAGGGAAGG
IL-18	CCAGCATCAGGACAAAGAAA	TACAGTGAAGTCGGCCAAAG
IGF-1	ACAGGCTATGGCTCCAGCAT	GCTCCGGAAGCAACACTCAT
IGF-2	CACGCTTCACTTTGTCTGTTCGG	CAGCACTCTTCCACGATGCCAC
Igfbp-2	CCAAGAAGCTGCGACCAC	GGGATGTGCAGGGAGTAGAG
Igfbp-3	GAGTGTGGAAAGCCAGGTTGTC	GGCATGGAGTGGATGGAACT
Igfbp-5	TCTAGCCATCCACACTGCTG	AAGAAAGCAAAGCGTTGGAA
Gapdh	TGCACCACCAACTGCTTAG	GGATGCAGGGATGATTGGC
Tbp	GCTCTGGAATTGTACCGCAGC	CTCTTGGCTCCTGTGCACAC
Binding site 1	AGAGCAGCTGCATTGATGTG	CGCTGTGGTATGCTAACTGG
Binding site 2	TAGATCACCCCCAACAATGG	CACCCACACATCAATGCAG
Binding site 3	CTGTGAAAAGGCAGGGCTAC	ATCAAGCGAGCAGAGAGTGG
Binding site 4	GGTGGTGTGCACCTATAGCC	CGAGGCCGCTCACTATAAAC
Binding site 5	CTGAGAGCTGGGGTGAGTG	TGAAACAGGGTCTCATGCAC
Binding site 6	TTTTTCCTCTGCCAGCGTTAG	AGAGGCTAAACTTTCCTTTTCCTC
Binding site 7	AAATTGTGGCTCCCCAGT	TGTCCTGTGGCTGTCCTATG
Binding site 8	TTAGTTTCCAAGCGGCTCTC	GCCCAGATGTTTTTGGTTTG
Binding site 9	AGAGGCAGGAGAAGCAGACC	AGGATGACCAGCCCTATGTG
Binding site 10	CTGTTCCTGGTCCTCACAGC	TCTGGGAAAAATGGATTTGC
Binding site 11	TGGACGTCAAGTCTGCTCTG	CTCTGCAAAAGAGGCTGTGG
Rar alpha	GGGAGTTTTTAAGCGCTGTGAG	GGAGCAGCTCACTTCCTACC
Negative control	GAAATTCTGTGTTGGCCGCA	TCAGCACCTACAATTCTGACCA
