# Supplementary material for: TG-interacting factor 1 (Tgif1)-deficiency attenuates bone remodeling and blunts the anabolic response to parathyroid hormone
Source: Nat Commun. 2019 Mar 22;10:1354. doi: 10.1038/s41467-019-08778-x (PMC6430773; doi:10.1038/s41467-019-08778-x)
Supplement: Supplementary file 2 — Description of Additional Supplementary Files [file 41467_2019_8778_MOESM2_ESM.pdf]

Description of Additional Supplementary Information

File Name: Source Data File

Description: The Source Data File contains uncropped images of the immunoblots presented in the main figures of the manuscript.
