# Supplementary material for: TG-interacting factor 1 (Tgif1)-deficiency attenuates bone remodeling and blunts the anabolic response to parathyroid hormone
Source: Nat Commun. 2019 Mar 22;10:1354. doi: 10.1038/s41467-019-08778-x (PMC6430773; doi:10.1038/s41467-019-08778-x)
Supplement: Supplementary file 3 — Reporting Summary [file 41467_2019_8778_MOESM3_ESM.pdf]

nature research

Corresponding author(s): Eric Hesse
Last updated by author(s): Jan 16, 2019

Reporting Summary

Nature Research wishes to improve the reproducibility of the work that we publish. This form provides structure for consistency and transparency in reporting. For further information on Nature Research policies, see Authors & Referees and the Editorial Policy Checklist.

Statistics

For all statistical analyses, confirm that the following items are present in the figure legend, table legend, main text, or Methods section.

n/a	Confirmed	
☐	☒	The exact sample size (*n*) for each experimental group/condition, given as a discrete number and unit of measurement
☐	☒	A statement on whether measurements were taken from distinct samples or whether the same sample was measured repeatedly
☐	☒	The statistical test(s) used AND whether they are one- or two-sided<br>*Only common tests should be described solely by name; describe more complex techniques in the Methods section.*
☒	☐	A description of all covariates tested
☐	☒	A description of any assumptions or corrections, such as tests of normality and adjustment for multiple comparisons
☐	☒	A full description of the statistical parameters including central tendency (e.g. means) or other basic estimates (e.g. regression coefficient) AND variation (e.g. standard deviation) or associated estimates of uncertainty (e.g. confidence intervals)
☐	☒	For null hypothesis testing, the test statistic (e.g. *F*, *t*, *r*) with confidence intervals, effect sizes, degrees of freedom and *P* value noted<br>*Give P values as exact values whenever suitable.*
☒	☐	For Bayesian analysis, information on the choice of priors and Markov chain Monte Carlo settings
☒	☐	For hierarchical and complex designs, identification of the appropriate level for tests and full reporting of outcomes
☒	☐	Estimates of effect sizes (e.g. Cohen's *d*, Pearson's *r*), indicating how they were calculated

Our web collection on statistics for biologists contains articles on many of the points above.

Software and code

Policy information about availability of computer code

Data collection	Putative Tgif binding sites in the SOST promoter were identified using the online platform ALGGEN-PROMO.
Data analysis	Excel, Prism, MAPRSeq v.1.2.1, TopHat 2.0.6, HTSeq, Sequest HT, SwissProt, SILAC quantitation: Event detector and precursor ion quantifier algorithms of Proteome Discoverer.

For manuscripts utilizing custom algorithms or software that are central to the research but not yet described in published literature, software must be made available to editors/reviewers. We strongly encourage code deposition in a community repository (e.g. GitHub). See the Nature Research guidelines for submitting code & software for further information.

Data

Policy information about availability of data

All manuscripts must include a data availability statement. This statement should provide the following information, where applicable:
- Accession codes, unique identifiers, or web links for publicly available datasets
- A list of figures that have associated raw data
- A description of any restrictions on data availability

Next generation RNA-sequencing data that support the findings of this study have been deposited at the Center for Biotechnology Information with the accession code GSE89132. The mass spectrometry proteomics data that support the findings of this study have been deposited to the ProteomeXchange Consortium via the PRIDE partner repository with the dataset identifier PXD012303.

Field-specific reporting

Please select the one below that is the best fit for your research. If you are not sure, read the appropriate sections before making your selection.

☒ Life sciences ☐ Behavioural & social sciences ☐ Ecological, evolutionary & environmental sciences

For a reference copy of the document with all sections, see nature.com/documents/nr-reporting-summary-flat.pdf

Life sciences study design

All studies must disclose on these points even when the disclosure is negative.

Sample size	No sample size calculations were performed but the number of mice analyzed in animal studies was determined in agreement with the standards in the field.
Data exclusions	All experimental animals that were alive by the time of analysis were included in the study. For all data points, an outlier test was performed.
Replication	Experiments were repeated at least three times as biological replicates with minimum of two technical replicates. All data could be replicated.
Randomization	Mice of the same genotype were randomized to the treatment or control group.
Blinding	Investigators were not blinded to the group allocation during the experiment, but assessment of the outcome by bone histomorphometry was performed in a blinded manner.

Reporting for specific materials, systems and methods

We require information from authors about some types of materials, experimental systems and methods used in many studies. Here, indicate whether each material, system or method listed is relevant to your study. If you are not sure if a list item applies to your research, read the appropriate section before selecting a response.

Materials & experimental systems

n/a	Involved in the study	
☐	☒	Antibodies
☐	☒	Eukaryotic cell lines
☒	☐	Palaeontology
☐	☒	Animals and other organisms
☒	☐	Human research participants
☒	☐	Clinical data

Methods

n/a	Involved in the study	
☒	☐	ChIP-seq
☒	☐	Flow cytometry
☒	☐	MRI-based neuroimaging

Antibodies

Antibodies used	anti-Tgif1: rabbit monoclonal, Abcam, Cat. No: ab52955; anti-Tgif2, rabbit polyclonal, Millipore, Cat. No: 09-718; anti-Sema3E, goat polyclonal, R&D Systems, Cat. No: AF3239; anti-pCREB, rabbit monoclonal, Cell Signaling, Cat. No: 87G3; anti-Mef2c, rabbit monoclonal, Abcam, Cat. No: 197070; anti-Actin, mouse monoclonal, Millipore, Cat. No: MAB1501; anti-rabbit or anti-mouse secondary antibodies, Promega, Cat. No: W401B, W402B; anti-Tgif1, Santa Cruz Biotechnology, Cat. No: sc9084; anti-IgG, Abcam, Cat. No: ab37415.
Validation	Antibodies were used according to the recommendation of the manufacturer. Antibodies had to yield a clear band of the predicted molecular weight. For some antibodies, further validation was performed in the laboratory using knockdown approaches of the protein of interest. If needed, dilutions were optimized.

Eukaryotic cell lines

Policy information about cell lines

Cell line source(s)	ST2: DSMZ (Cat. No.: ACC 333), UMR-106: ATCC (Cat. No.: CRL-1661), OCY454 from Dr. Paola Divieti Pajevic, IDG-SW3 from Dr. Lynda Bonewald
Authentication	Cell lines used in this study have not been authenticated.
Mycoplasma contamination	All cell lines have been regularly tested negative for mycoplasma contamination.
Commonly misidentified lines (See ICLAC register)	No cell line used is listed in the database of commonly misidentified cell lines maintained by ICLAC

Animals and other organisms

Policy information about studies involving animals; ARRIVE guidelines recommended for reporting animal research

Laboratory animals	Mus musculus, Tgif1+/-, Tgif1 loxp/+, Osx-Cre+, Dmp1-Cre+, PTHR1loxp/+, Dmp1-caPTHR1, C57Bl/6 background, males + females, 8-12 weeks old.
Wild animals	The study did not involve wild animals.
Field-collected samples	Mice were housed and maintained in the research animal facility of the research institution. Mice had free access to water and food, were kept at ambient temperature with a 12h day/night cycle.
Ethics oversight	The study received approval by the local authority for animal welfare.

Note that full information on the approval of the study protocol must also be provided in the manuscript.
