# Supplementary material for: TG-interacting factor 1 (Tgif1)-deficiency attenuates bone remodeling and blunts the anabolic response to parathyroid hormone
Source: Nat Commun. 2019 Mar 22;10:1354. doi: 10.1038/s41467-019-08778-x (PMC6430773; doi:10.1038/s41467-019-08778-x)
Supplement: Supplementary file 4 — Source Data [file 41467_2019_8778_MOESM4_ESM.pdf]

Figure 2f

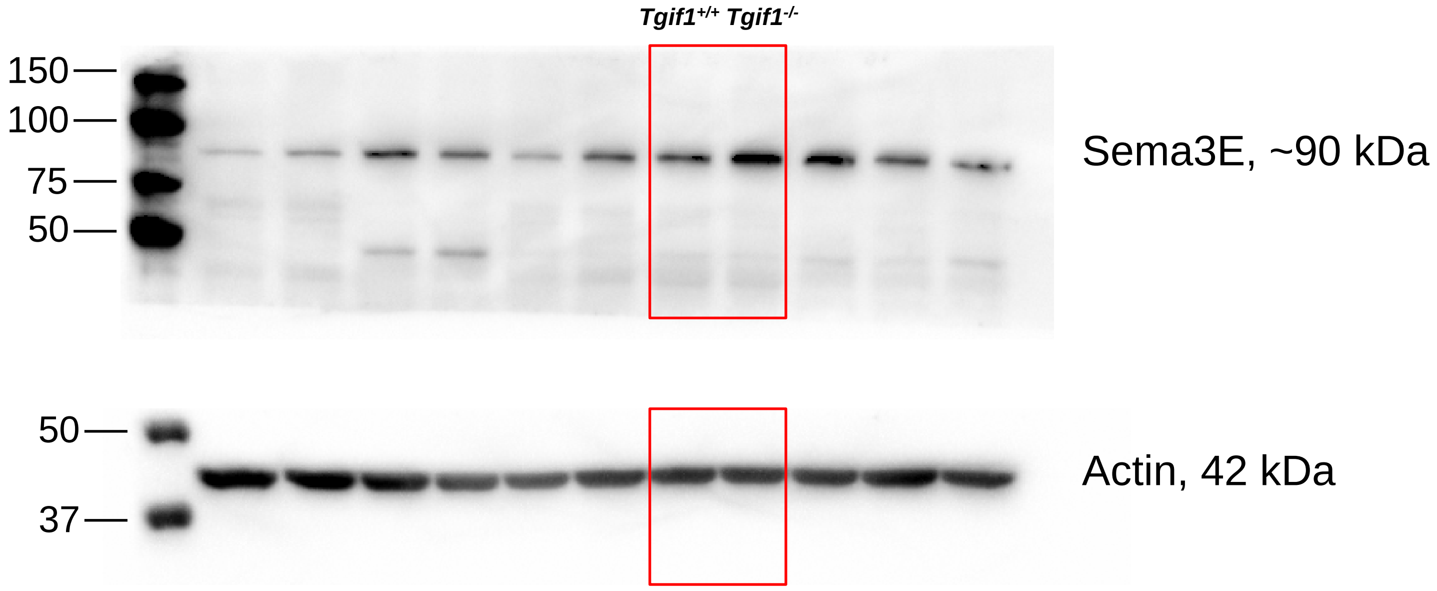

Source data to Fig. 2f Uncropped immunoblot of Semaphorin 3E (Sema3E) protein expression in calvarial osteoblasts obtained from *Tgif1*$^{+/+}$ and *Tgif1*$^{-/-}$ mice. Immunoblot for actin was used as a loading control. Molecular weight in kilo Dalton (kDa) is indicated.

Figure 3a

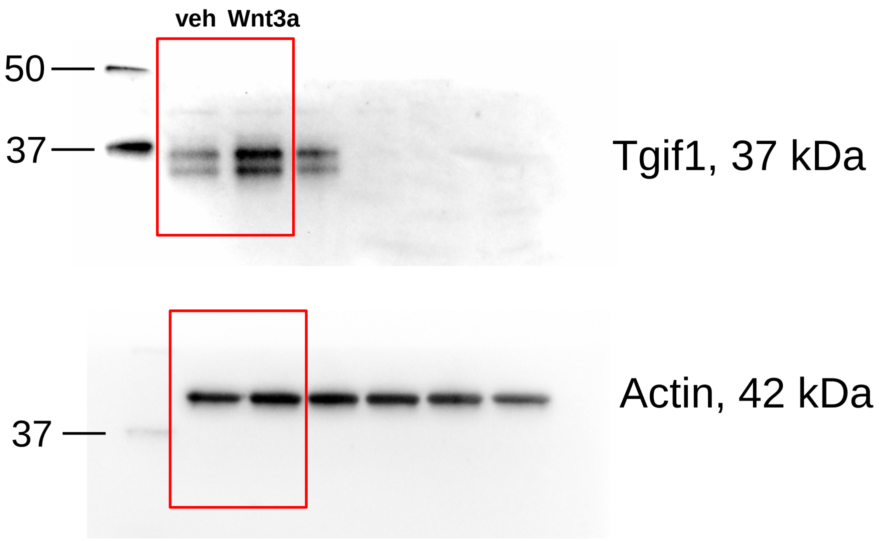

Source data to Fig. 3a Uncropped immunoblot of Tgif1 protein expression in wild-type calvarial osteoblasts after stimulation with recombinant Wnt3a or vehicle (veh) for 4 hours. Immunoblot for actin was used as a loading control. Molecular weight in kilo Dalton (kDa) is indicated.

Figure 5a

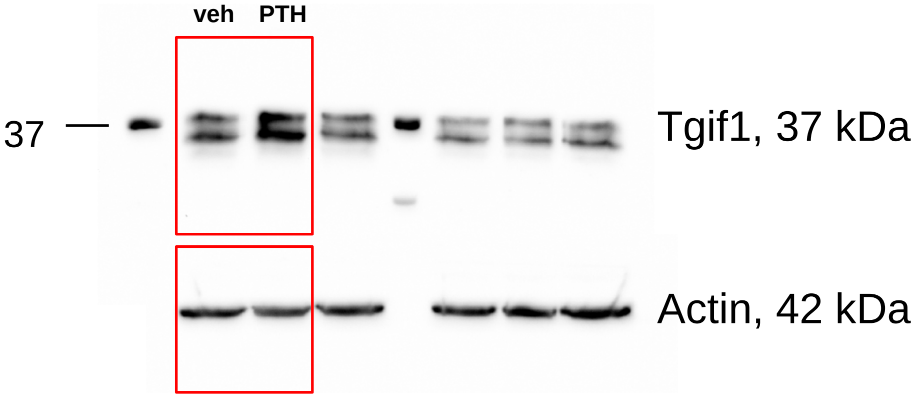

Source data to Fig. 5a Uncropped immunoblot of Tgif1 expression in osteoblasts upon PTH or vehicle (veh) treatment. Immunoblot for actin was used as a loading control. Molecular weight in kilo Dalton (kDa) is indicated.

Figure 5e

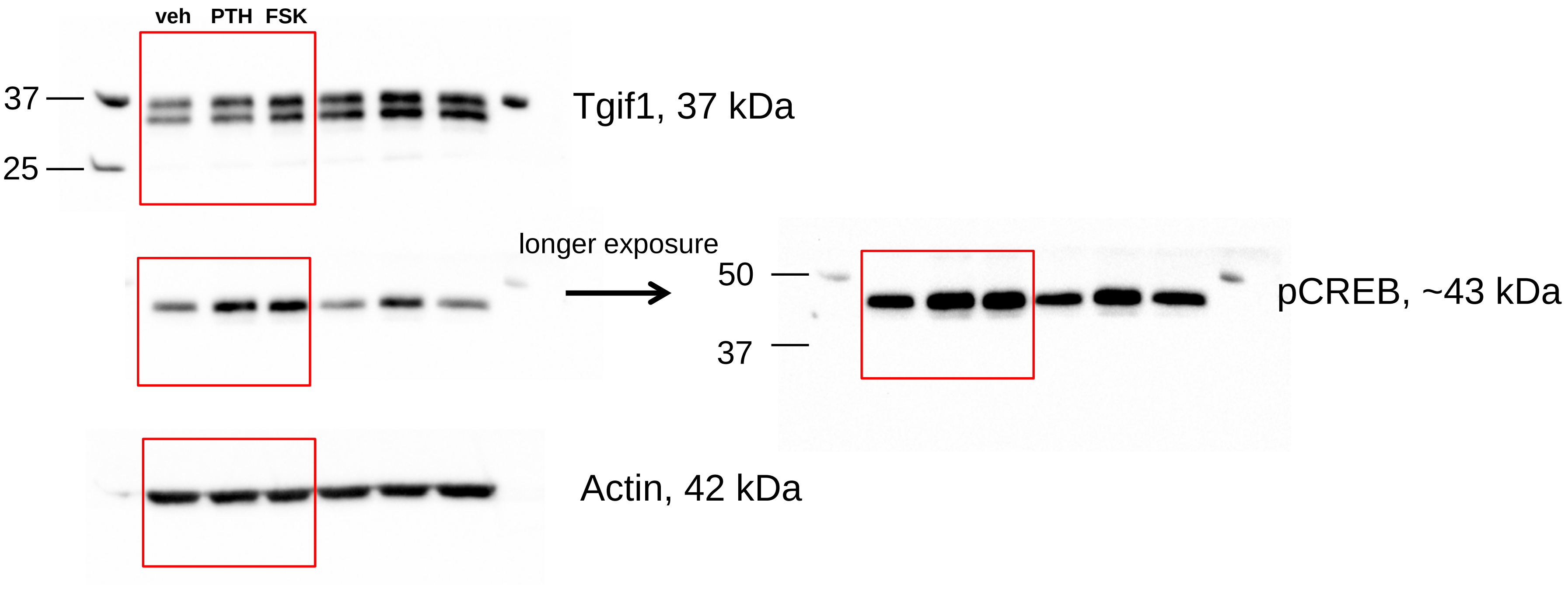

Source data to Fig. 5e Uncropped immunoblot of Tgif1 and pCREB in osteoblasts after stimulation with PTH, Forskolin (FSK) or vehicle (veh). A longer exposure of the immunoblot showing the abundance of pCREB is shown on the right. Immunoblot for actin was used as a loading control. Molecular weight in kilo Dalton (kDa) is indicated.

Figure 7e

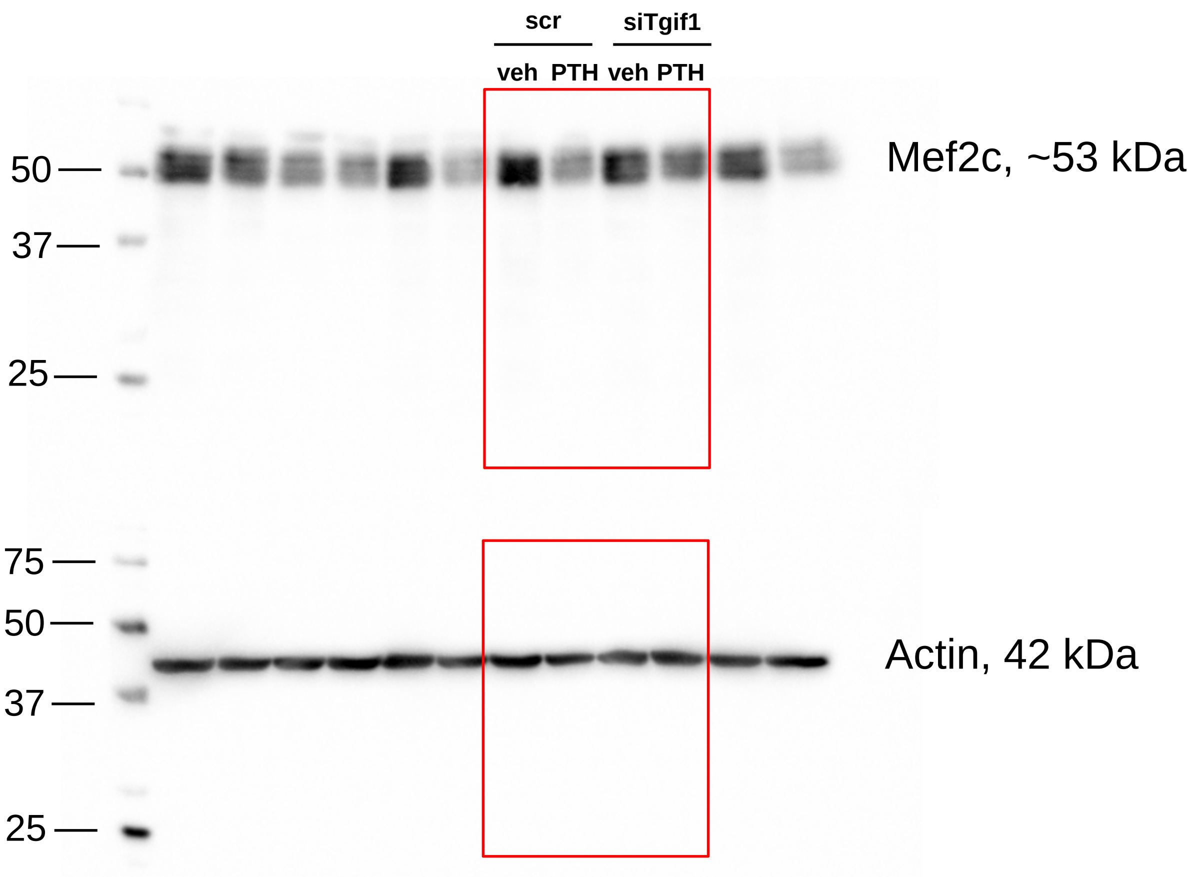

Source data to Fig. 7e Uncropped immunoblot demonstrating the protein abundance of Mef2c in UMR-106 cells. Cells were transfected with scrambled (scr) control siRNA or siRNA targeting Tgif1 (siTgif1). After 48 hours, cells were stimulated with PTH or vehicle (veh) for 8 hours. Immunoblot for actin was used as a loading control. Molecular weight in kilo Dalton (kDa) is indicated.
